# Intra‐subtype heterogeneity shapes treatment response in *KMT2A*‐rearranged ALL across all age groups

**DOI:** 10.1002/hem3.70324

**Published:** 2026-02-19

**Authors:** Alina M. Hartmann, Lorenz Bastian, Malwine J. Barz, Johannes Haas, Eric Amelunxen, Patrick Ehm, Lennart Lenk, Michaela Kotrova, Thomas Beder, Fabio D. Steffen, Kerstin Rauwolf, Nadine Wolgast, Sonja Bendig, Cecilia Bozzetti, Julia Alten, Mayukh Mondal, Annika Rademacher, Julia Heymann, Wencke Walter, Claudia Haferlach, Aeint‐Steffen Ströh, Anke K. Bergmann, Thomas Burmeister, Nicola Gökbuget, Beat Bornhauser, Jean‐Pierre Bourquin, Monika Brüggemann, Martin Schrappe, Gunnar Cario, Claudia D. Baldus

**Affiliations:** ^1^ Medical Department II, Hematology and Oncology University Medical Center Schleswig‐Holstein, Campus Kiel Kiel Germany; ^2^ University Cancer Center Schleswig‐Holstein (UCCSH) University Medical Center Schleswig‐Holstein Kiel Germany; ^3^ Clinical Research Unit CATCH ALL KFO 5010 Kiel Germany; ^4^ Division of Pediatric Oncology and Children's Research Center University Children's Hospital Zurich Zurich Switzerland; ^5^ Department of Pediatrics I, Pediatric Hematology/Oncology University Medical Center Schleswig‐Holstein, Campus Kiel Kiel Germany; ^6^ Institute for Clinical Molecular Biology, University Medical Center Schleswig‐Holstein, Campus Kiel Kiel Germany; ^7^ MLL Munich Leukemia Laboratory Munich Germany; ^8^ Clinical Genetics and Genomic Medicine University Hospital Würzburg Würzburg Germany; ^9^ Department of Hematology, Oncology and Cancer Immunology CVK, Charité – Universitätsmedizin Berlin, Corporate Member of Freie Universität Berlin and Humboldt‐Universität zu Berlin Berlin Germany; ^10^ Department of Medicine II, Hematology/Oncology Goethe University Hospital Frankfurt am Main Germany

## Abstract

*KMT2A*‐rearranged B‐cell acute lymphoblastic leukemia (*KMT2A*r B‐ALL) exhibits significant heterogeneity in age of onset, developmental origins, and clinical outcomes. The interplay of individual factors influencing early treatment response within this high‐risk molecular subtype remains poorly elucidated. To identify determinants of early treatment response to induction chemotherapy, we analyzed 465 *KMT2A*r B‐ALL cases spanning a wide age range (1 month to 89 years) by integrating transcriptomic and genomic profiling with functional drug response and measurable residual disease (MRD) kinetics. We observed a strong inverse correlation between MRD clearance with advancing age (P = 2.1E−04), proximity to early B‐cell‐precursor developmental state (low maturity score, P = 1.3E−03), and *AFF1* as fusion partner (P = 7.0E−04). A multivariable analysis confirmed the strong impact of maturity (P = 0.02) and *KMT2A* fusion partner (P = 0.03) on MRD clearance, supporting the concept that the cell's developmental state defines therapy response. Gene expression analysis identified cellular traits that relate to MRD clearance (e.g., chromatin organization, immune modulation, and proliferation). This gene expression classifier grouped cases not only by MRD clearance but also by ex vivo sensitivity to induction therapy drugs. Notably, good responders to ex vivo induction drugs were characterized by a higher maturity score (P = 1.8E−03), whereas for less mature *KMT2A*r B‐ALL cases, response profiles suggested higher Venetoclax sensitivity. Our study provides an integrative framework linking developmental phenotype, fusion partner, and MRD kinetics across the full age spectrum of *KMT2A*r B‐ALL. These insights may support future risk‐adapted strategies and therapeutic targeting, particularly in immature *KMT2A*r B‐ALL.

## INTRODUCTION

*KMT2A*‐rearranged B‐cell acute lymphoblastic leukemia (*KMT2A*r B‐ALL) is considered a high‐risk molecular subtype due to poor treatment response and high risk of relapse. *KMT2A* is the most prevalent genomic driver in infant ALL, accounting for ~70% of the cases,^1^, ^2^ yet also occurs in older children (~2%–5%^2^, ^3^) and adult patients (~15%^4^). The prognosis is especially poor for infant patients and older adults, suggesting intra‐subtype molecular heterogeneity to account for the differences in treatment response. Over 90 *KMT2A* fusion partners have been identified with over 80% of *KMT2A*r cases having an *AFF1*, *MLLT1*, or *MLLT3* rearrangement.^5^ Adult cases predominantly carry *KMT2A::AFF1* rearrangements (*AFF1*r); pediatric and infant cases display a greater variety of fusion partners. *KMT2A* fusion partners have shown to impact therapy response and outcome with *MLLT3*r cases showing superior outcome compared to *AFF1*r in infant patients, but not in pediatric patients (age > 1 year).^2^, ^6^ Molecular characterization of an infant *KMT2A*r B‐ALL cohort revealed subclusters associated with different driver fusions,^7^ suggesting different mechanisms based on the underlying gene fusion partner.

In addition to the heterogeneity of molecular drivers, molecular B‐ALL subtypes have differential proximity to the physiological developmental stages of normal B‐cell development, from hematopoietic stem cells (HSCs) to committed pre‐B cells. Age‐overriding immunoglobulin rearrangement profiling across molecular subtypes revealed a higher immunogenetic maturity in pediatric patients, compared to adults,^8^, ^9^ and single‐cell‐sequencing studies of ALL found increasing cell plasticity with age.^10^ However, molecular characterization of *KMT2A*r B‐ALL cases has unraveled an immature leukemic origin in younger patients^10^, ^11^, ^12^ associated with increased cell plasticity, retained latent myeloid potential, and co‐expression of myeloid marker genes.^10^ This immature, high plasticity phenotype has been shown to impact drug sensitivity,^13^, ^14^ necessitating alternative treatment strategies. As the bispecific T‐cell engager monoclonal antibody, blinatumomab, chimeric antigen receptor T‐cell therapy, and novel agents such as BH3‐mimetic Venetoclax and Menin inhibitors^15^, ^16^ are being incorporated into novel treatment algorithms, future investigations will have to assess their efficacy in the light of the underlying leukemic origin of the ALL.

To address the multi‐faceted intra‐subtype heterogeneity in an age‐overarching cohort and to explore the impact of the underlying cellular context in disease and therapy response, we conducted a multi‐omics analysis in a large cohort of 465 *KMT2A*r diagnostic B‐ALL cases spanning a unique age range upon diagnosis (1 month to 89 years), and including common *KMT2A* fusions with representative frequencies.^5^ We demonstrate that the maturity score (reflecting proximity to B‐cell developmental stages), the *KMT2A* fusion partner, and the age at diagnosis impact measurable residual disease (MRD) clearance in *KMT2A*r ALL. A machine‐learning model revealed maturity and fusion partners as stronger predictors for MRD than age. Importantly, distinct leukemic regulatory programs are linked to treatment response and thus may provide a basis for understanding and targeting molecular mechanisms of drug resistance in *KMT2A*r B‐ALL.

## MATERIALS AND METHODS

### Patients and transcriptome data set

**Figure 1:**
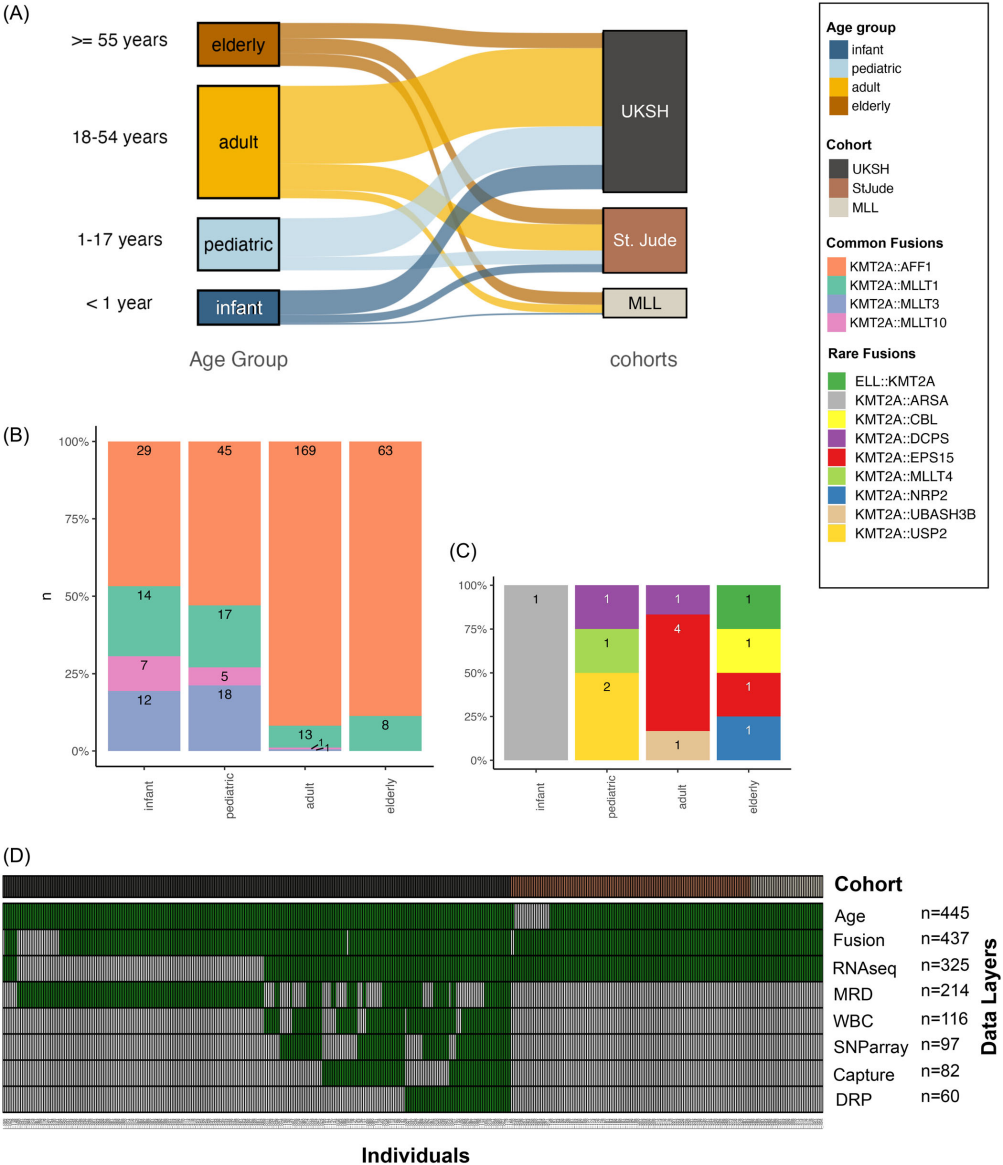
**Age‐overriding KMT2A‐rearranged B‐cell acute lymphoblastic leukemia (KMT2Ar B‐ALL) patient cohort. (A)** Included patients (*n* = 465) of different age groups (*n* = 445/465 with available age information: ≥55 *n* = 79, 18–54 *n* = 207, 1–17 *n* = 96, and <1 *n* = 63) from three cohorts (UKSH *n* = 288, St. Jude^17^ *n* = 136, and MLL^18^ *n* = 41). **(B)** Distribution of common *KMT2A* fusions across age groups (*n* = 422, 96.6%). **(C)** Distribution of rare *KMT2A* fusions (*n* = 15, 3.4%). **(D)** Overview of available data layers per patient (see also Supporting Information S1: Table 1) by cohort (gray: internal discovery cohort, brown: St. Jude cohort,^17^ and beige: Munich Leukemia Laboratory cohort^18^). Available layers are marked by black columns: fusion partner known, age known, RNAseq counts, measurable residual disease (MRD) at two time points available, white blood cell count at diagnosis (WBC) available, virtual karyotypes by single nucleotide polymorphism (SNP) array, DNA capture, drug response profiling (DRP).

We have aggregated a unique cohort of *n* = 465 *KMT2A*r ALL cases spanning the whole age range (1 month to 89 years, Figure 1A) including the most common *KMT2A* fusion partners with representative frequencies^5^ (Figure 1B,C). The cohort is well characterized with available omics layers including RNAseq (*n* = 325), Sanger sequencing/RNAseq for gene fusion detection (*n* = 436), single nucleotide polymorphism (SNP) arrays (*n* = 135), lymphoid capture panel DNA‐sequencing^19^ (*n* = 82), and drug response profiling^20^, ^21^ (DRP, *n* = 61). Moreover, data on clinical annotations including age (*n* = 445), genomic *IG‐R* rearrangement profile (*n* = 47), white blood cell count at diagnosis (WBC, *n* = 223), and longitudinal MRD monitoring (*n* = 214) were available. For the *n* = 325 RNAseq cases, *n* = 148 were uniformly sequenced in our facility and defined as the discovery cohort. The remaining *n* = 177 cases (validation cohort) were from publicly available sources^17^ (*n* = 133) and from a collaborating laboratory^18^ (*n* = 41); (Figure 1A,D; Supporting Information S1: Table 1).

### Multi‐omics data

Transcriptomic data analysis,^22^ fusion gene detection, and DNA capture panel sequencing^19^ were performed by common standard (Supporting Information S2: Supplementary Methods). DRP was performed in an established co‐culture system as previously described^20^, ^21^, ^23^ (Supporting Information S2: Supplementary Methods).

### Therapy response data

For patients treated according to the AIEOP‐BFM ALL/INTERFANT and GMALL study group protocols, MRD measurements were performed in the central reference laboratories using clonal IG/TR or *KMT2A* rearrangements as previously described.^24^ Pediatric‐inspired induction therapy protocols in adults with comparable MRD timepoints in pediatric regimens allowed for common kinetic definitions of MRD clearance. We defined two early MRD assessment timepoints at which patients of all age groups had received comparable treatment regimens according to the respective protocols (Supporting Information S3: Figure 1A). Timepoint 1 (TP1) corresponds to “MRD End of Induction” in the pediatric protocol (main components: Glucocorticoids, Daunorubicin, Vincristine, and Asparaginase) and to “before Induction II” in the adult treatment protocol. Timepoint 2 (TP2) corresponded to “MRD End of consolidation” (main components: Cyclophosphamide, Cytarabine, and 6‐Mercaptopurine) in the pediatric protocol and to “post Induction II” (main components: Cyclophosphamide, Cytarabine, 6‐Mercaptopurine, and Asparaginase) in the adult protocol. Thus, we classified MRD evaluable patients based on MRD kinetics into “Fast MRD clearance” if MRD was negative or positive below the quantifiable range at TP1 and negative at TP2. “Slow MRD clearance” was defined as positive MRD (>10^−4^) at TP2. Patients with positive MRD at TP1 and negative MRD or positive MRD below the quantifiable range at TP2 were defined “intermediate” (Supporting Information S3: Figure 1B, Supporting Information S1: Table 5).

### Data analysis and statistics

#### Gene expression

The MRD gene expression signature was defined using an ordinal regression model to account for the structured nature of MRD categories (Supporting Information S2: Supplementary Methods).

To validate gene sets, we performed unsupervised clustering of normalized gene counts across our discovery cohort and, where applicable, the validation cohort, with the R pheatmap package (Version 1.0.12). Biological function was annotated using Gene Ontology (GO) enrichment analysis (Supporting Information S2: Supplementary Methods).

#### Maturity score

We calculated for each patient sample a maturity score, representing the transcriptional proximity to corresponding physiological B‐cell developmental states. As a healthy reference, we used previously published transcriptional profiles of seven fluorescence‐activated cell sorting‐sorted B‐cell populations from four healthy adult bone marrow donors, representing the early B‐cell developmental trajectory from HSC via pro‐B, pre‐B‐I, and pre‐B‐II stages until the immature B‐cell stage.^25^ The stage‐specific gene sets are implemented in the machine‐learning classifier ALLCatchR,^25^ which computes *z*‐scaled enrichment scores for each of the cell‐type stages based on individual bulk transcriptomes (Supporting Information S1: Table 2).

**Figure 2:**
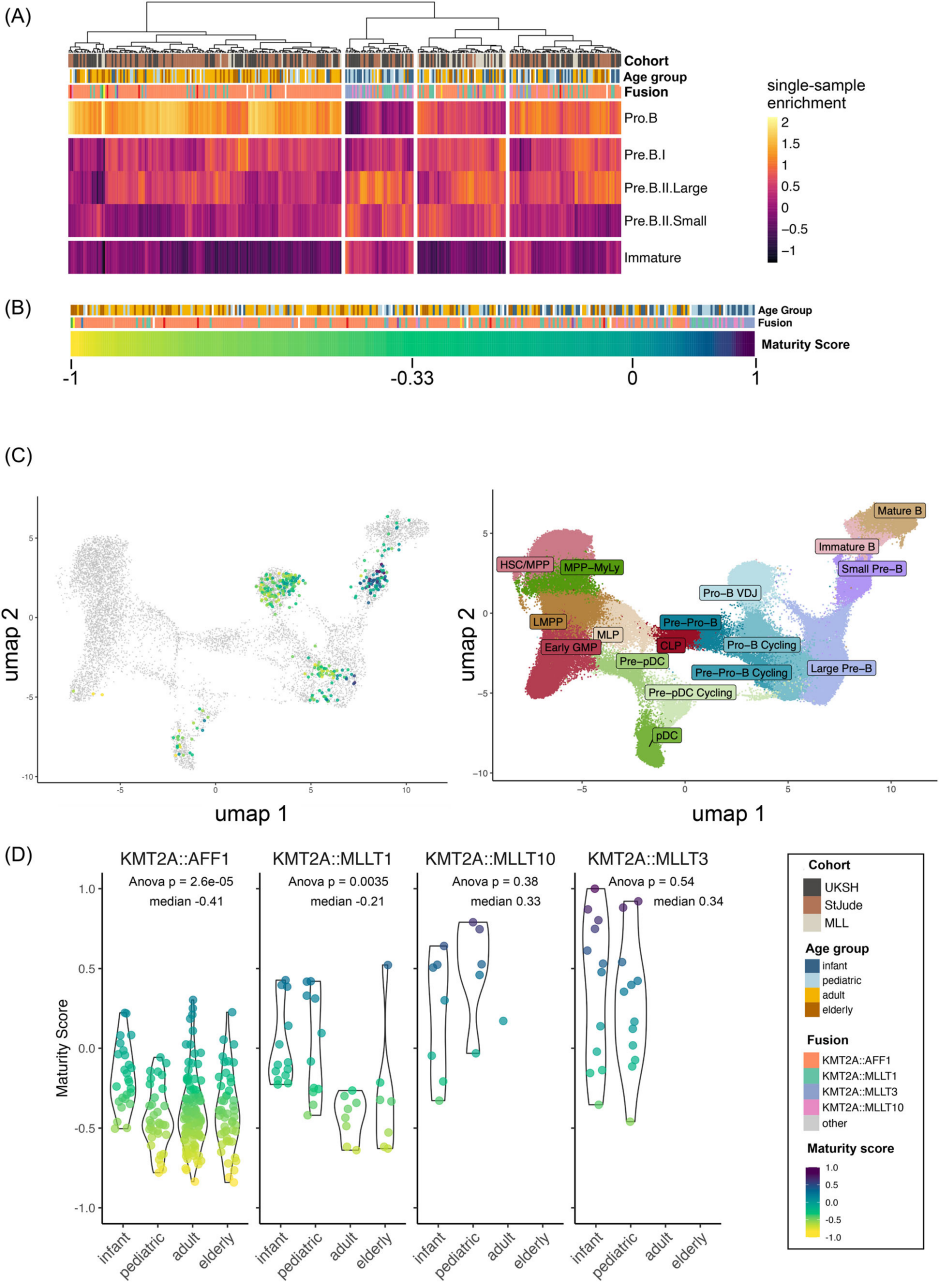
**Transcriptional maturity score in KMT2A‐rearranged acute lymphoblastic leukemia (KMT2Ar ALL) correlates with age and fusion partner. (A)** Gene set enrichment analysis of *n* = 325 *KMT2A*r ALL cases. Enrichment score represents proximity to five healthy B‐cell‐precursor developmental stages (pro‐B, pre‐B‐I, pre‐B‐II‐large, pre‐B‐II‐small, and immature‐B), calculated based on the RNAseq data using ALLCatchR tool.^25^ **(B)** Developmental trajectories of *KMT2A*r ALL were condensed into a single maturity score ranging from −1 to 1 by fitting linear regression to the enrichment scores in (A) and calculating the slope. Low maturity scores represent immature transcriptional developmental state and proximity to pro‐B cells, high maturity scores represent more mature (pre‐B‐II, immature‐B) transcriptional developmental states. **(C)** *KMT2A*r samples were mapped to the expression matrix of the human single‐cell B‐cell developmental atlas^10^ and colored by maturity score (left). Reference cell states are annotated in the right panel. Samples with low maturity score map to early cell stages (pro‐B‐V(D)J/pre‐pro‐B cycling), samples with higher maturity score map to mature B‐cell stages (pre‐B/immature‐B). **(D)** Maturity score distribution between age groups within driver fusion groups (AFF1r *n* = 221, MLLT1r *n* = 43, MLLT10r *n* = 14, and MLLT3r *n* = 28). Multi‐comparison analysis of variance (ANOVA) between age groups within each fusion group reveals significantly different maturity scores between age groups in AFF1r (P = 2.6E−05) and MLLT1r (P = 0.0035) cases.

To get an estimate of each patient's transcriptional maturity from this profile, we calculated relative distances of each of these cell stages from the healthy donor population in a principal component analysis (PCA) (Supporting Information S3: Figure 2A), creating a linear axis from immature to more mature cell stages. Each patient sample then had an estimate for its proximity to each of the cell stages on this axis (Supporting Information S1: Table 3; Supporting Information S3: Figure 2B, three exemplary cases). To quantify these dynamics, we used the relative cell‐type distances from the PCA as numerical values along an *x*‐axis and the enrichment scores per cell stage as corresponding *y*‐values and computed the slope of the linear regression along the developmental trajectory. For example, very immature cases would have high proximity to earlier cell stages and low proximity to later cell stages, represented by a downward slope along the B‐cell developmental stages (Supporting Information S3: Figure 2B top panel). More mature cases are represented by higher scores for later B‐cell stages and low scores for early stages, represented by an upward slope along the cell stages (Supporting Information S3: Figure 2B bottom panel).

The scaled slope represents the maturity score, with high positive values representing proximity to more mature B‐cell stages and negative values representing proximity to immature cell stages (Supporting Information S3: Figure 2C,D).

**Figure 3:**
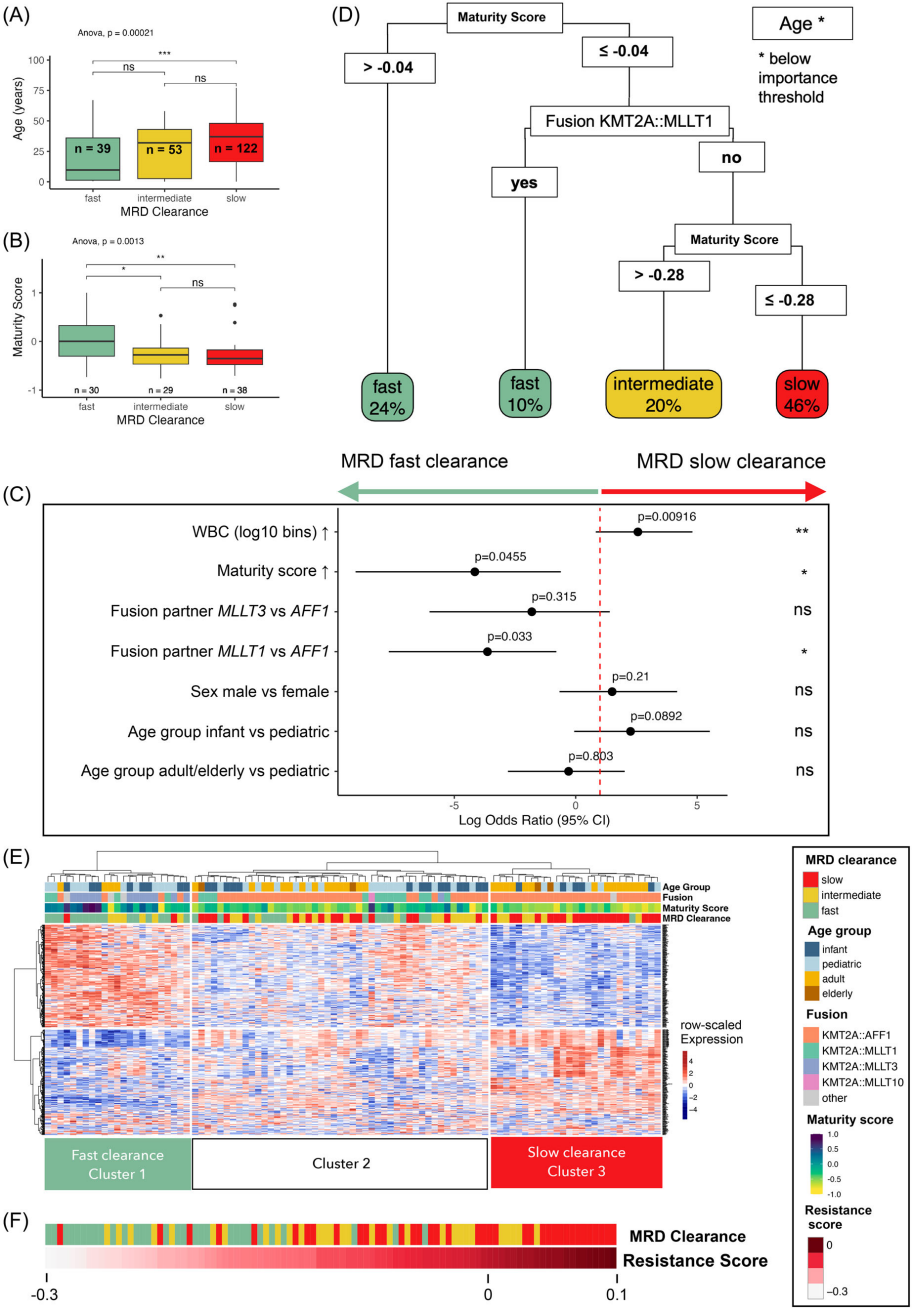
**Measurable residual disease (MRD) clearance is modulated by age, gene fusion, and maturity score. (A)** Distribution of age within MRD clearance categories (MRD fast clearance: *n* = 39, median age: 9.4 years; MRD intermediate clearance: *n* = 53, median age: 32 years; and MRD slow clearance: *n* = 122, median age: 37 years). Age is significantly higher in MRD slow clearance patients (multi‐comparison analysis of variance [ANOVA], P = 0.00021). **(B)** Distribution of maturity scores within MRD‐clearance categories (MRD fast clearance: *n* = 30; MRD intermediate clearance: *n* = 29; and MRD slow clearance: *n* = 38). Maturity scores are significantly higher in MRD fast clearance patients (multi‐comparison ANOVA, P = 0.0013). **(C)** Multivariable logistic regression results. The model tested the effect of white blood cell count at diagnosis (WBC), maturity score, fusion partner (*MLLT3* and *MLLT1* compared to *AFF1* as reference, respectively), sex, and age group (infant and adult/elderly compared to pediatric as reference). *x*‐Axis shows log odds ratio and 95% confidence intervals, P‐values are annotated for each factor (WBC: P = 0.00916, maturity score: P = 0.0455, fusion *MLLT3*: P = 0.315, fusion *MLLT1*: P = 0.033, sex: P = 0.21, age group infant: P = 0.0892, and age group adult/elderly: P = 0.803). **(D)** Machine‐learning trained decision tree, trained to predict MRD‐clearance categories based on age, fusion, and maturity score. The highest level split predicts patients with a maturity score >−0.04 to have fast clearance, the second split predicts patients with a maturity score ≤−0.04 and MLLT1r to have fast MRD clearance, the third split predicts patients with maturity scores between >−0.28 and −0.04 into intermediate MRD clearance, and patients with maturity scores ≤−0.28 and no MLLT1r to have slow MRD clearance. **(E)** Gene expression signature detected from a proportional odds linear regression model grouping patients by MRD clearance. Cluster 1 is enriched for patients with fast MRD clearance (Cluster 1: *n* = 12/18 [67%] fast, *n* = 5/18 [28%] intermediate, and *n* = 1/18 [6%] slow; Cluster 3: *n* = 1/28 [4%] fast, *n* = 7/28 [25%] intermediate, and *n* = 20/28 [71%] slow; chi‐squared P < 0.0001). **(F)** Resistance score (total score from single sample gene set enrichment analysis using genes upregulated in Cluster 3 as up‐set and genes upregulated in Cluster 1 as down‐set).

**Figure 4:**
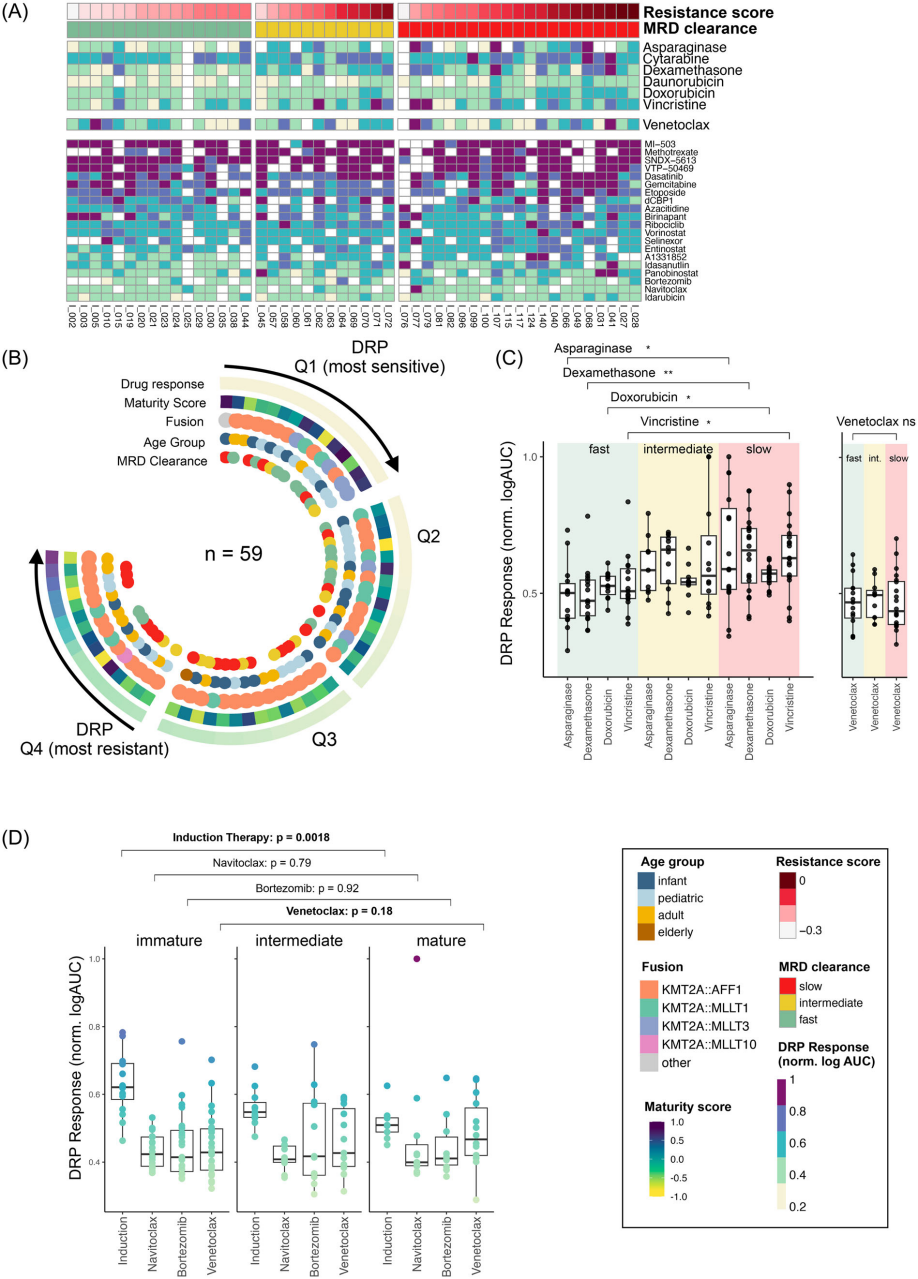
**Ex vivo drug response to induction therapy drug mirrors slow measurable residual disease (MRD) clearance.** Drug response of ex vivo *KMT2A*r samples (*n* = 61). **(A)** normalized log2 area under the curve (norm.logAUC) for 27 compounds with at least *n* = 40 cases measured.^20^, ^21^ Top six rows represent induction therapy drugs. Patients were grouped according to MRD clearance (*n* = 49) and annotated with resistance score from Figure 3F. **(B)** Circos plot of patients (*n* = 59) ordered and separated into quartiles by their mean response (norm.logAUC) to induction phase drugs, and annotated with maturity score, fusion partner, age group, and MRD clearance. Quartiles 1 and 2 (Q1/Q2, *n* = 15, respectively; most sensitive) were enriched for fast MRD clearance, high maturity score, non‐AFF1r, and pediatric age. Quartiles 3 and 4 (Q3/Q4, *n* = 15, respectively; most resistant) were enriched for slow MRD clearance, low maturity score, AFF1r, and older age (chi‐squared tests: MRD clearance P = 0.003, maturity score group P = 0.064, fusion P = 0.153, and age group P = 0.025, Supporting Information S3: Figure 7). **(C)** Response (norm.logAUC) to induction therapy drugs was poorer in patients with slow MRD clearance (*n* = 17; red box) compared to patients with fast MRD clearance (*n* = 16; green box; mean norm.logAUC across drugs: 0.61 [slow MRD] vs. 0.52 [fast MRD], Wilcox‐test per drug: Asparaginase P = 0.02, Dexamethasone P = 0.0027, Doxorubicin P = 0.041, Vincristine P = 0.012, and Cytarabine and Daunorubicin not significant). **(D)** Response to induction phase drugs (left) and Venetoclax (right). Patients grouped by maturity score group (cutoffs −0.04 and −0.28 based on cutoffs from trained decision tree (Figure 3D). Patients in the lowest maturity score group (≤−0.28) are significantly more resistant to patients in the highest maturity score group (>−0.04) (Wilcox‐test, P = 0.0018). The effect is inverse for Venetoclax (Wilcox‐test P = 0.18). DRP, drug response profiling.

To validate our maturity score, we mapped our cohort to two recently published single‐cell atlases of physiological B‐cell development^10^, ^26^ and additionally correlated it to the B‐cell map author's multipotency score^10^ (Supporting Information S2: Supplementary Methods, Supporting Information S3: Figures 3A–C and 4).

#### Clonal evolution by IG‐R characterization

Amplicon‐based next‐generation sequencing (NGS) with EuroClonality‐NGS IGH‐VJ‐FR1 and IGH‐DJ primers and the EuroClonality‐NGS central in‐tube quality/quantification control and the detection of clonally evolving DNJ‐stems was detected as described before.^9^ Briefly, the “DNJ‐stem” of nucleotide junctions was screened for similarity. Clonotypes sharing the same stem were considered clonally related and underwent further analysis. To decide whether the stem should be considered stable or evolving, amplicon‐seq results were filtered for low read numbers, potential artefacts (highly similar clonotypes), and the structure of the locus as described before.

#### Multivariable analysis on MRD clearance

To quantify how age, maturity, and gene fusion affect MRD clearance, we applied two complementary multivariable methods. First, we used logistic regression to model the effect of WBC (log10 bins), the maturity score, fusion gene partner (reference: *KMT2A::AFF1*), sex (reference: female), and age group (reference: pediatric) to calculate odds ratios for each factor given the other factors. Using drop‐1‐chi‐sq tests (comparing the full model to each 1‐factor reduced model), we quantified the contribution and respective statistical robustness for every factor.

In a second approach, we trained a machine‐learning decision tree to find the hierarchical importance of maturity score, age, and fusion partner (Supporting Information S2: Supplementary Methods). The model did not have high enough predictive capacity to be used as an MRD‐clearance classification tool (combined accuracy 56.7%, compared to 27.9% random assignment). However, feature importance values could be extracted to learn which features contribute most to the decision process.

## RESULTS

To disentangle the molecular underpinnings of treatment response in *KMT2A*r B‐ALL, we aggregated a cohort of *n* = 465 initial diagnosis samples, including *n* = 325 RNAseq samples from own (*n* = 148) and external (*n* = 177)^17^, ^18^ sequencing, representing three clinical cohorts and an age spectrum from 1 month to 89 years (Figure 1A). Patients were categorized for subsequent analyses as infants (<1 year; *n* = 63), pediatric (1–17 years, *n* = 96), adult (18–54 years, *n* = 207), and elderly patients (≥55 years, *n* = 79). To avoid batch effects, we processed *n* = 148 samples homogeneously in‐house (patient age: 2 months to 79 years, median 13 years), serving as a discovery cohort. Integrative data analysis comprised genomic profiling for karyotypes (SNP array), single‐nucleotide variants (capture panel sequencing^19^), and IG/TR rearrangement status, transcriptomic profiling for gene expression and gene fusions and functional DRP.

### *KMT2A* fusion partners are associated with age

Among B‐ALL molecular subtypes, *KMT2A*r ALL is characterized by the widest age range and holds a diverse repertoire of *KMT2A* fusion partner genes. In line with studies in large cohorts,^5^ we observed a significant correlation between fusion partners and patient's age (Figure 1B; *n* = 305, Chi‐sq. test P < 0.0001). While *AFF1* was the most frequent fusion partner gene (*n* = 221/321; 69%), it was strongly enriched in adult/elderly patients (71% vs. infant/pediatric 48%; P = 7.7E−12). Pediatric patients showed more diverse fusion partners, with *MLLT3*r exclusively found in infants/pediatric cases, and *MLLT1*r and *MLLT10*r enriched in infant/pediatric cases (61% and 92% vs. adult/elderly 8% and 0.6%, P = 0.008 and P = 1.5E−4, respectively). Rare *KMT2A* fusion partners were also included in our cohort (*EPS15* [*n* = 5], *USP2* [*n* = 2], and *DCPS* [*n* = 2], one of each: *MLLT4*, *UBASH3B*, *NRP2*, *ARSA*, *CBL*, and *ELL*, Figure 1C), providing a comprehensive representation of *KMT2A*r ALL.

Unsupervised clustering of the top 1000 most variably expressed genes across our discovery cohorts revealed a notable influence of the gene fusion partner on the gene‐expression‐based patient clustering. To account for this, we have characterized the fusion‐specific gene expression profiles (Supporting Information S2: Supplementary Methods, Supporting Information S1: Table 4) and included the gene fusion partner as a covariate in subsequent statistical models (Supporting Information S3: Figure 6).

### *KMT2A*r ALLs are characterized by few cooperating genomic aberrations

Previous studies have found few cooperating genomic events in *KMT2A*r ALL.^3^ To investigate the landscape of cooperating genomic events in our cohort, we performed capture panel sequencing for genes recurrently involved in ALL (*n* = 82) and SNP arrays (*n* = 104). We detected pathogenic mutations in 32 of the 82 analyzed samples (in 8/26 [31%] pediatric patients and in 24/56 [43%] adult patients). In total, 21 of 82 patients (26%) carried either a KRAS or NRAS mutation; the remaining mutations were found in TP53 (6%), ATM (5%), CDKN2B (2%), and CXCR4, FAT1, RUNX1, and TCF3 (<2%, Supporting Information S3: Figure 7A). The SNP arrays revealed diploid karyotypes in nearly all cases, without patterns of recurrent chromosomal gains and losses (Supporting Information S3: Figure 7B). Focal deletions were not detected with sufficient robustness by our applied methods and were therefore not systematically evaluated.

### *KMT2A*r ALL in adults is enriched for immature transcriptional developmental states

To systematically characterize the developmental underpinnings of *KMT2A*r ALL, we made use of our immuno‐genomic defined RNAseq reference of human B‐lymphopoiesis.^25^ Single sample gene set enrichment analysis (ssGSEA) for gene sets defining normal B‐lymphopoiesis was used to define the proximity of individual *KMT2A*r ALL samples to their physiological B‐cell counterparts (Figure 2A). Regression analysis across enrichment scores was used to condense values into a single maturity score (Figure 2B, Supporting Information S3: Figure 2A–C, the Materials and Methods section).

We validated the stage of differentiation by correlating *IGH* rearrangement patterns on a genomic level^9^ in *n* = 47 patients (*n* = 14 infant, *n* = 13 pediatric, and *n* = 20 adult): most patients displayed complete *IGH* rearrangements, indicative of late pro‐B to pre‐B cells of origin. In line with previous findings,^9^ the majority of cases revealed clonally evolving IGH‐stems, and the absence of clonal evolution correlated with higher maturity scores (Supporting Information S3: Figure 5). In addition, we mapped bulk‐RNAseq data to two single‐cell atlases of human B‐cell development^10^, ^26^ confirming the prediction that samples with low maturity scores corresponded to either the pro‐B V(D)J compartment or pro‐B cycling cells, whereas samples with high maturity scores aligned with more mature B‐cell stages (large pre‐B, small pre‐B, and immature B, Figure 2C and Supporting Information S3: Figure 4). We calculated the “multipotency score” postulated by Iacobucci et al.^10^ for our cohort and found a strong correlation with our maturity score (*R* ^2^ = 0.54, P < 0.001, Supporting Information S3: Figure 3A) and accordingly, an increase in the maturity score and a decrease in the multipotency score with predicted B‐developmental stages based on the B‐cell developmental map (Supporting Information S3: Figure 3B,C, respectively).

Increasing maturity correlated with younger age (Spearman's rank correlation, *R* = 0.45, P < 2.2E−15). To separate the effect of age on maturity from the effect of the fusion gene, we assessed the maturity scores separately for every driver fusion (Figure 2D). *AFF1*r cases were most immature (*n* = 221, median maturity score = −0.41) compared to samples carrying other fusions. Within *AFF1*r cases, we observed increasing immaturity with increasing age (*R* = 0.16, P = 0.024); however, the effect was small and was mainly driven by the infant age group. *MLLT1*r cases had a higher median maturity score than *AFF1*r cases (*n* = 43, median maturity score = −0.21; P = 5.3E−06) and increasing age correlated with increasing immaturity (R = 0.6, P = 4.8E−05). *MLLT3*r and *MLLT10*r had the highest maturity scores (*MLLT3*r*: n* = 28, median maturity score = 0.33; *MLLT10*r*: n* = 14, median maturity score = 0.34) and were only observed in young patients (median age 0.95 and 0.9 years, respectively, Figure 2D).

### Age, driver fusion, and maturity modulate MRD clearance

To define the molecular underpinnings of treatment response, we made use of our integrated age‐overriding cohort including infants (*n* = 31, 14.5%), pediatric (*n* = 49, 22.9%), adult (*n* = 113, 52.8%), and elderly (*n* = 21, 9.8%) patients treated according to BFM‐based pediatric or adult treatment protocols with corresponding MRD measurements available. MRD was measured at two timepoints during induction therapy and after induction/before consolidation. Of the 214 MRD evaluable patients, 18.2%, 24.8%, and 57.0% had a fast, intermediate, and slow MRD clearance, respectively. Age significantly correlated with the initial MRD clearance (*n* = 214, multi‐comparison analysis of variance [ANOVA] P = 0.0002), with the median age of patients in fast MRD responders being 9.6 years in contrast to 37 years in slow responders (Figure 3A). Overall, 51% of infant (*n* = 16/31), 31% of pediatric (*n* = 15/49), 65% of adult (*n* = 73/113), and 86% of elderly (*n* = 18/21) patients showed slow MRD clearance.

When stratified by fusion, *AFF1*r cases predominantly displayed intermediate or slow MRD clearance (*n* = 133/144; 92%) compared to *MLLT1*r (*n* = 13/23; 57%) and *MLLT3*r (*n* = 9/18; 50%) cases (total *n* = 185, chi‐squared test with Monte Carlo simulation, P = 0.0005; Supporting Information S3: Figure 8A). Correspondingly, infants showed faster MRD clearance if they were *MLLT1*r or *MLLT3*r, but slower in the case of *AFF1*r (*MLLT1*r*/MLLT3*r: 6/13 slow/intermediate; *AFF1*: 17/17 slow/intermediate, Fisher's exact test, P = 0.0008). Similarly, lower maturity scores were associated with poorer MRD clearance (Wilcox P = 0.0025; Figure 3B).

To disentangle these interrelated effects, we performed a multivariable logistic regression including maturity score, *KMT2A* fusion partner, age group, sex, and WBC count (Figure 3C). In this model, high WBC was associated with slow MRD clearance (Log‐OR 2.56, P = 0.0091), and higher maturity scores were associated with fast MRD clearance (Log‐OR −4.15, P = 0.046). Independently, *MLLT1*r cases also showed a higher likelihood of fast clearance compared to *AFF1*r cases (Log‐OR −3.63, P = 0.033). The overall contribution of each covariate, quantified by drop‐one likelihood‐ratio tests (Supporting Information S3: Figure 8B), revealed that WBC explained the largest share of model deviance, followed by fusion partner and maturity score, although the effect of the maturity score had a lower P‐value (P = 0.019). Age group lost significance (P = 0.089) when these variables were included, indicating that the apparent age effect is largely driven by underlying differences in transcriptional maturity and fusion partner rather than chronological age itself.

As a complementary intuitive approach, we trained a decision tree (Figure 3D, the Materials and Methods section). The tree grouped patients first by maturity score (>−0.04 to fast), next by fusion (*MLLT1*r to fast), and the remaining cases by maturity score (≤−0.28 to slow, >−0.28 to intermediate), mirroring the results from the regression model.

### MRD‐driven gene expression signatures

To characterize the molecular programs underlying MRD clearance, we used RNAseq expression data and built an ordinal regression model, with age and gene fusion as covariates for MRD‐associated genes independent of these factors. This resulted in a 448‐gene signature (Supporting Information S1: Table 6) which grouped patients into three main clusters in an unsupervised clustering approach (Figure 3E). Two clusters distinguished patients with fast and slow MRD clearance (Cluster 1; *n* = 15/23 [65%] fast clearance, Cluster 3; *n* = 26/27 [96%] intermediate/slow clearance, Fisher's exact test P = 0.007; Figure 3E). We condensed the gene signature into a single “resistance score” using ssGSEA and confirmed that this score was predictive for MRD clearance (Figure 3F).

There was only a minimal overlap of this MRD‐gene expression signature with gene expression profiles associated with *KMT2A* fusion partner (Supporting Information S1: Table 4, Supporting Information S3: Figure 9A) or defining different B‐cell stages (Supporting Information S3: Figure 9B), underscoring that the MRD signature represented specific MRD‐associated molecular programs irrespective of the fusion signature.

To functionally annotate the signature of MRD‐slow‐responders, we applied gene set enrichment analysis for genes upregulated in MRD‐slow‐responders (Supporting Information S2: Supplementary Methods, Supporting Information S3: Figure 9C) and found enrichment of GO‐terms associated with chromatin organization, driven by genes coding for core histones (H1 linker histones *H1‐3* and *H1‐4*; H2B histones [e.g., *H2BC6*, *H2BC11*]; H2A histones [e.g., *H2AC16*], and H3 histones). Groups 2 and 4 were functionality associated with cell differentiation and proliferation, respectively. The fifth GO‐term group was associated with immune response and leukocyte activation. Accordingly, it was driven by immune‐modulator genes like *IL5RA*, *IL1RL1*, and *AZU1* and inflammatory response genes like *ANXA1*. Overall, the MRD‐associated gene regulation appears not to be driven by one prominent signaling pathway but rather reflects a complex interplay of cellular functions related to proliferation and differentiation, which in turn points to the developmental underpinnings (maturity) as a modulator of treatment sensitivity.

### Age, maturity, and gene fusion reflect ex vivo drug response

To functionally characterize the therapy response, we performed ex vivo DRP on diagnostic primary patient material. We screened 61 *KMT2A*r cases (*n* = 21 infant, *n* = 22 pediatric, *n* = 16 adult, and *n* = 1 elderly) with a library of *n* = 27 drugs, including both standard‐of‐care chemotherapeutics and targeted therapy agents such as BCL2‐family inhibitors Venetoclax and Navitoclax, proteasome‐inhibitor Bortezomib, or HDAC‐inhibitor Panobinostat (Figure 4A).

We split samples into quartiles based on their mean drug response to induction phase drugs (Asparaginase, Cytarabine, Dexamethasone, Daunorubicin, Doxorubicin, and Vincristine) quantified by the logarithmic area under the dose response curve (logAUC, Figure 4B). We observed high concordance with MRD clearance: samples with a good ex vivo drug response (Q1 and Q2) were significantly enriched for patients with fast MRD clearance (Q1/2 sensitive DRP group included 13/26 fast MRD‐clearance patients compared to the Q3/4 DRP resistant group with only 2/22 fast MRD‐clearance patients; Fisher's exact test P = 0.004, Supporting Information S3: Figure 10). This difference in response was also significant for the single drugs Dexamethasone, Daunorubicin, Asparaginase, and Vincristine individually (Wilcox‐test slow vs. fast MRD clearance; P‐value cutoff 0.1; Dexamethasone P = 0.0027; Daunorubicin P = 0.084, Vincristine P = 0.012, and Asparaginase P = 0.02, Figure 4C).

Q1 and Q2 (most DRP sensitive) were slightly enriched for infant and pediatric patients, although not significantly (Fisher's exact test P = 0.16), significantly enriched for non‐*AFF1* gene fusion partners (Fisher's exact test P = 0.01), and had significantly higher maturity scores (Wilcox‐test, P = 0.016; Figure 4B and Supporting Information S3: Figure 10).

In line with these results, our gene‐expression‐based resistance score (Figure 3F), reflecting high expression of MRD‐slow‐clearance associated genes, was significantly higher in cases with poor response to induction phase drugs (Q1/2: median resistance score −0.13, Q3/4: median resistance score −0.03, Wilcox‐test P = 0.002). Thus, the activation of MRD‐associated gene regulation at diagnosis can be captured by gene expression and is reflected in functional ex vivo DRP (Figure 4A).

We also evaluated three Menin inhibitors (MI‐503, SNDX‐5613, and VTP‐50469). In contrast to our main DRP panel, these compounds were assessed after 6 rather than 3 days of incubation to account for their slower kinetics. Within the *KMT2A*r cohort, we observed no significant variation in Menin inhibitor sensitivity with respect to developmental maturity, age, or MRD clearance (Supporting Information S3: Figure 11). For MI‐503, AFF1‐rearranged cases showed a slightly reduced response compared to other fusions (ANOVA P = 0.02), although the effect size was modest and not observed for SNDX‐5613 or VTP‐50469. Because the assay used a different incubation time, these data could not be integrated with the rest of the DRP panel, and we therefore cannot compare overall Menin inhibitor response to other drugs or to different molecular subtypes.

### Venetoclax responders are more frequently immature and characterized by AFF1 fusions

In addition to standard induction chemotherapy drugs, we further analyzed targeted compounds revealing several drugs with notable activities in our cohort, including the proteasome‐inhibitor Bortezomib (*n* = 45), the HDAC‐inhibitor Panobinostat (*n* = 57), and the BCL2‐inhibitors Navitoclax (*n* = 46) and Venetoclax (*n* = 58). We matched the sensitivity profiles for these targeted compounds with MRD clearance and maturity to find potential vulnerabilities for MRD‐slow responding patients.

By comparing drug response with maturity scores (based on the cutoffs defined in the decision tree in Figure 3D: maturity score ≤−0.28 [immature]; ≤−0.04 and >−0.28, >−0.04 [mature]), we did not observe a significant increase in sensitivity to Navitoclax, Bortezomib, or Venetoclax with increasing maturity, as opposed to induction therapy drugs (Figure 4D). However, for Venetoclax, we noticed a slight inverse trend showing the highest sensitivity in the most immature group, although this trend was not significant (Wilcox‐test, P = 0.18). Consistently, we did not observe a correlation of MRD clearance with Venetoclax response (Figure 4C). In fact, cases sensitive to Venetoclax mirror the MRD‐slow‐clearance profile, with low maturity scores, predominantly *AFF1*r and older age (Supporting Information S3: Figure 10), highlighting the potential of Venetoclax in the treatment of cases with an MRD‐slow‐clearance profile, especially those with low B‐cell maturity.

## DISCUSSION

Prognosis of *KMT2A*r ALL varies dramatically across age groups, suggesting an intra‐subtype molecular heterogeneity to account for the differences in treatment response. To address the underlying biology, we have aggregated the—to our knowledge—largest cohort of *KMT2A*r patients extensively characterized by multi‐level data analysis and clinical annotations. This is the first study to integrate transcriptomic developmental state, driver fusion partner, longitudinal MRD clearance, and ex vivo drug profiling across the full age spectrum of *KMT2A*r B‐ALL. By linking these molecular layers to treatment outcome, we identify cellular vulnerabilities and provide a framework for risk stratification.

Previous studies^7^, ^10^, ^11^, ^12^, ^13^, ^14^ on *KMT2A*r ALL have mostly focused on single aspects like age,^13^ infant patients,^7^, ^11^, ^12^ or included only small cohorts of *KMT2A*r patients.^11^, ^14^ Because *KMT2A* fusion partners are unevenly distributed across age groups and influence molecular heterogeneity and outcome, large data sets are necessary to account for these contributing factors.^1^, ^2^, ^6^ In addition, as only a few cooperating genomic events characterize the *KMT2A*r subgroup, it is likely that cell intrinsic factors drive its intra‐subtype heterogeneity.

Consistent with prior reports,^1^, ^2^, ^4^, ^5^, ^6^ age correlated with early MRD clearance. However, our fine‐scale dissection demonstrates that fusion partner and maturity correlate with age and thus a complex interplay of different factors predicts MRD clearance. Notably, our analysis revealed that the transcriptional maturity score emerged as a central predictor of early treatment response. Patients with slow MRD clearance had significantly lower maturity scores than fast responders, and this association remained independently predictive even after accounting for age and fusion partner. In fact, maturity and fusion together emerged as stronger predictors of MRD clearance than age alone. This aligns with recent reports that less differentiated (“adult‐like”) ALL phenotypes tend to be chemotherapy‐resistant (e.g., mercaptopurine resistance in pediatric *KMT2Ar* ALL^13^ and asparaginase resistance in pre‐pro‐B‐ALL with high BCL2 expression^14^), reinforcing that developmental state influences drug sensitivity. Consistent with single‐cell analyses of B‐ALL,^10^ our bulk‐transcriptome maturity score strongly correlated with the previously published “multipotency score,” validating that our metric captures developmental immaturity. Importantly, using bulk RNAseq makes this assessment feasible in a clinical setting. Beyond the maturity score, we also derived an MRD‐clearance gene expression signature that segregated slow versus fast responders, highlighting upregulated pathways of chromatin organization, proliferation, and immune modulation in slow responders. Our study expands existing analyses to the largest *KMT2A*r cohort, integrating fusion‐specific effects, full age range, gene expression, and direct MRD and ex vivo response correlations and demonstrating that developmental maturity, together with fusion status, defines early therapy response.

Functional ex vivo drug profiling mirrored clinical MRD clearance for drugs administered during the initial therapy protocol in line with previous studies,^27^, ^28^ further advocating the potential use of ex vivo induction therapy response for risk stratification.^29^ Integrating our DRP data with the transcriptional profiling revealed that poor response to standard‐of‐care induction chemotherapeutics is associated with immature transcriptional states. Accordingly, the MRD‐slow‐clearance cases were characterized by an immature developmental stage, *AFF1*r, and poorer ex vivo response to standard‐of‐care chemotherapeutics. Intriguingly, cases that were responding best to Venetoclax matched the profile of MRD‐slow clearance, with immature developmental stage and predominantly *AFF1*r. Thus, patients predicted to have poor chemotherapy response (low maturity score, *AFF1*r) might benefit from alternative therapies like Venetoclax, a hypothesis supported by our data, though it warrants clinical validation.

We provide the analytical groundwork to explore interdependencies between transcriptional developmental B‐cell state, selection of genomic drivers, gene regulation, and biological and clinical phenotypes in B‐ALL across molecular subtypes. Among the molecular correlates we examined, the maturity score stood out as the most clinically relevant indicator of early treatment response. In contrast, the 448‐gene MRD expression signature we identified, while insightful for biology, should be viewed as exploratory and would require further validation before clinical use.

In the future, a consensus definition of B‐ALL proximity to normal B‐lymphopoiesis might be included in novel risk stratification models, pending prospective validation in clinical trials, and putatively extending beyond *KMT2A*r B‐ALL.

## AUTHOR CONTRIBUTIONS

**Alina M. Hartmann**: Conceptualization; investigation; funding acquisition; writing—original draft; visualization; formal analysis; data curation; methodology; software; supervision; project administration. **Lorenz Bastian**: Conceptualization; investigation; funding acquisition; writing—original draft; formal analysis; methodology; supervision; visualization. **Malwine J. Barz**: Investigation; writing—review and editing; formal analysis; conceptualization; methodology; visualization. **Johannes Haas**: Investigation; writing—review and editing; formal analysis; software. **Eric Amelunxen**: Investigation; writing—review and editing; formal analysis. **Patrick Ehm**: Writing—review and editing; investigation; resources; methodology. **Lennart Lenk**: Investigation; writing—review and editing. **Michaela Kotrova**: Software; investigation; writing—review and editing. **Thomas Beder**: Software; investigation; writing—review and editing. **Fabio D. Steffen**: Software; investigation; formal analysis; writing—review and editing. **Kerstin Rauwolf**: Investigation; writing—review and editing. **Nadine Wolgast**: Software; writing—review and editing. **Sonja Bendig**: Resources; writing—review and editing. **Cecilia Bozzetti**: Resources; writing—review and editing. **Julia Alten**: Resources; validation; writing—review and editing. **Mayukh Mondal**: Software; writing—review and editing; resources. **Annika Rademacher**: Resources; writing—review and editing. **Julia Heymann**: Resources; writing—review and editing. **Wencke Walter**: Resources; writing—review and editing. **Claudia Haferlach**: Resources; writing—review and editing. **Aeint‐Steffen Ströh**: Resources; writing—review and editing. **Anke K. Bergmann**: Supervision; writing—review and editing. **Thomas Burmeister**: Resources; writing—review and editing. **Nicola Gökbuget**: Supervision; resources; writing—review and editing; data curation. **Beat Bornhauser**: Investigation; methodology; supervision; writing—review and editing. **Jean‐Pierre Bourquin**: Investigation; supervision; writing—review and editing. **Monika Brüggemann**: Supervision; resources; writing—review and editing; data curation. **Martin Schrappe**: Resources; writing—review and editing; supervision. **Gunnar Cario**: Conceptualization; data curation; investigation; supervision; funding acquisition; project administration; writing—review and editing. **Claudia D. Baldus**: Conceptualization; investigation; data curation; supervision; funding acquisition; project administration; writing—review and editing.

## CONFLICT OF INTEREST STATEMENT

Burmeister: *Pfizer Inc.:* honoraria. Haferlach: *MLL Munich Leukemia Laboratory*: equity ownership. Gökbuget: *Amgen*, *AstraZeneca*, *Autolus*, *Clinigen*, *Gilead*, *Incyte*, *Jazz Pharmaceuticals*, *Novartis*, *Pfizer*, *Sanofi*, and *Servier:* consultancy, honoraria, other: Advisory board; *Amgen*, *Clinigen*, *Incyte*, *Jazz Pharmaceuticals*, *Novartis*, *Pfizer*, and *Servier:* research funding. Brüggemann: *Amgen*, *Becton Dickinson*, *AstraZeneca*, *Jazz*, and *Pfizer:* consultancy, honoraria, research funding, and speakers bureau. Schrappe: *JazzPharma*, *Servier*, and *Amgen:* honoraria, research funding, and speakers bureau. Cario: *Jazz Pharmaceuticals:* Other: travel support. Baldus: *Janssen*, *Astellas*, *Pfizer*, *AstraZeneca*, *Servier*, and *BMS:* consultancy, honoraria.

## ETHICS STATEMENT

This study was approved by the Ethik‐Kommission der medizinischen Fakultät der Christian‐Albrechts‐Universität zu Kiel, approval number D 416/21. The study was conducted in accordance with the principles of the Declaration of Helsinki.

## FUNDING

This study was funded in part by the Deutsche Forschungsgemeinschaft (DFG; German Research Foundation) project number 444949889 (KFO 5010 Clinical Research Unit “CATCH ALL” to A.M.H., L.B., M.J.B, P.E., L.L., N.W., S.B., M.M., A.R., M.B., M.S., G.C, and C.D.B.). Open Access funding enabled and organized by Projekt DEAL.

## Supporting information

Supporting Information.

Supporting Information.

Supporting Information.

## Data Availability

RNAseq count data are accessible from Zenodo at https://doi.org/10.5281/zenodo.15638437 for our cohort or through the original publications (St. Jude^17^ and MLL^18^). DRP normalized AUC data are provided in Supporting Information S1: Table 7. RNAseq raw fastq files will be provided from the corresponding author upon reasonable request.
