# Supplementary material for: Intra‐subtype heterogeneity shapes treatment response in KMT2A‐rearranged ALL across all age groups
Source: Hemasphere. 2026 Feb 19;10(2):e70324. doi: 10.1002/hem3.70324 (PMC12931199; doi:10.1002/hem3.70324)
Supplement: Supplementary file 2 — Supporting Information. [file HEM3-10-e70324-s002.docx]

**Supplementary Methods**

**Library preparation and sequencing of discovery cohort**

RNA was extracted from primary bone marrow aspirates or blood with at least 20% blasts at initial diagnosis and sequenced according to published protocols(1). Gene counts were normalized in R (Version 4.3.0) using the package DGEobj.utils (Version 1.0.6) to normalized log2 counts per million.

**Fusion gene detection**

Gene fusions were detected by PCR (n = 85) or from the transcriptome data using the fusioncatcher tool (Version 1.33).

**DNA capture sequencing**

Single nucleotide variations were called from genomic capture panels. Variants were annotated using variant effect predictor and filtered for pathogenic somatic mutations.

**Drug response profiling**

Leukemia cells were seeded on an hTERT-immortalized mesenchymal stromal monolayer and incubated with a compound library encompassing both FDA-approved, clinically actionable chemotherapeutics as well as single agents targeting specific signaling pathways and molecular targets (ABL/Src, BCL2, proteasome, HDAC, menin). Cells were exposed to the drugs in a six-point serial dilution for 72h before reading out their viability by fluorescence microscopy (Operetta, Perkin Elmer) using CyQuant as a live-cell marker. Viable cells were counted per condition and normalized to DMSO. The drug responses were fitted to a three-parameter dose-response model (logEC_50_, *E*_max_, slope parameter *b*) using the *drc* R package (Ritz et al. PlosOne, 2015) and integrated to yield the area-under-the-curve (AUC) as a drug scoring metric for estimating sensitivity or resistance(2,3).

**MRD gene expression signature**

The MRD gene expression signature was defined using an ordinal regression model to account for the structured nature of MRD categories. The model was calculated in R using the proportional odds logistic regression model in the MASS package (Version 7.3-60.0.1). For each coding gene, we calculated the effect of gene expression on the MRD category including the driver fusion and age as variables in the model. Significance scores were calculated by comparing the model t-values for the effect of gene expression on MRD to the normal distribution and log2-fold-changes which were calculated between MRD categories “fast” and “slow”. Genes were filtered for p-values smaller or equal to 0.05 and a log2-fold-change ≧1 (Supplementary Table S6).

**Gene set enrichment analysis**

MRD signature

To characterize the genes that were associated with slow MRD clearance, we extracted the genes upregulated in the respective gene expression cluster and searched for enrichment in functional GO-terms. We next filtered for genes most abundant in cell functional modules (present in at least 10 GO-terms) and for GO-terms including at least five genes from the list. We grouped genes and the respective enriched GO-terms which lead to five GO-term groups that represent diverse functionalities of the cell.

For validation, we mapped our bulk RNA-seq cohort to two single-cell bone marrow atlases of physiological B-cell development(5,6). We used the BoneMarrowMap R package(7) to predict most likely cell stage per bulk sample from the respective data sets and to map bulk samples to the single cell reference.

**Impact of fusion, immaturity and age on MRD clearance**

Decision Tree

with 10-fold cross validation and a maximum tree depth of five. We tested robustness across five different seeds.

1. Bastian L, Hartmann AM, Beder T, Hänzelmann S, Kässens J, Bultmann M, et al. UBTF::ATXN7L3 gene fusion defines novel B cell precursor ALL subtype with CDX2 expression and need for intensified treatment. Leukemia. 2022 June;36(6):1676–80.

2. Frismantas V, Dobay MP, Rinaldi A, Tchinda J, Dunn SH, Kunz J, et al. Ex vivo drug response profiling detects recurrent sensitivity patterns in drug-resistant acute lymphoblastic leukemia. Blood. 2017 Mar 16;129(11):e26–37.

3. Saorin A, Dehler A, Galvan B, Steffen FD, Ray M, Lu D, et al. Transcriptional remodeling shapes therapeutic vulnerability to necroptosis in acute lymphoblastic leukemia. Blood J. 2025 May 13;blood.2025028938.

4. Beder T, Hansen BT, Hartmann AM, Zimmermann J, Amelunxen E, Wolgast N, et al. The Gene Expression Classifier ALLCatchR Identifies B-cell Precursor ALL Subtypes and Underlying Developmental Trajectories Across Age. HemaSphere. 2023 Sept;7(9):e939.

5. Zeng AGX, Iacobucci I, Shah S, Mitchell A, Wong G, Bansal S, et al. Single-cell Transcriptional Atlas of Human Hematopoiesis Reveals Genetic and Hierarchy-Based Determinants of Aberrant AML Differentiation. Blood Cancer Discov. 2025 Apr 28;OF1–18.

6. Iacobucci I, Zeng AGX, Gao Q, Garcia-Prat L, Baviskar P, Shah S, et al. Multipotent lineage potential in B cell acute lymphoblastic leukemia is associated with distinct cellular origins and clinical features. Nat Cancer [Internet]. 2025 June 27 [cited 2025 July 25]; Available from: https://www.nature.com/articles/s43018-025-00987-2

7. Zeng AGX. _BoneMarrowMap: Single cell reference mapping onto Bone Marrow Hematopoiesis_. 2024.
