# Supplementary material for: Intra‐subtype heterogeneity shapes treatment response in KMT2A‐rearranged ALL across all age groups
Source: Hemasphere. 2026 Feb 19;10(2):e70324. doi: 10.1002/hem3.70324 (PMC12931199; doi:10.1002/hem3.70324)
Supplement: Supplementary file 3 — Supporting Information. [file HEM3-10-e70324-s001.docx]

**Supplementary Figures**

**S1. Treatment schema and MRD clearance classification.** **A**: Induction phase I and II for adult patients (GMALL study group) and pediatric patients (AIEOP-BFM study group) is depicted. **B**: Log levels of MRD measured at the respective reference laboratories using NGS-protocol. All patients had >10% blasts at diagnosis (n=214). Timepoint (TP1) 1 corresponded to “post Induction I” (adult) and post protocol 1A (ped.) respectively (total n=204, of which n=31 infants, n=48 pediatric, n=108 adult and n=18 elderly). Timepoint 2 (TP2) corresponded to “post Induction II”/”pre consolidation I” (adult) and post protocol 1B (ped) respectively (total n=205, of which n=31 infant, n=47 pediatric, n=106 adult, n=20 elderly). Resulting in n=214 patients evaluable for MRD clearance.


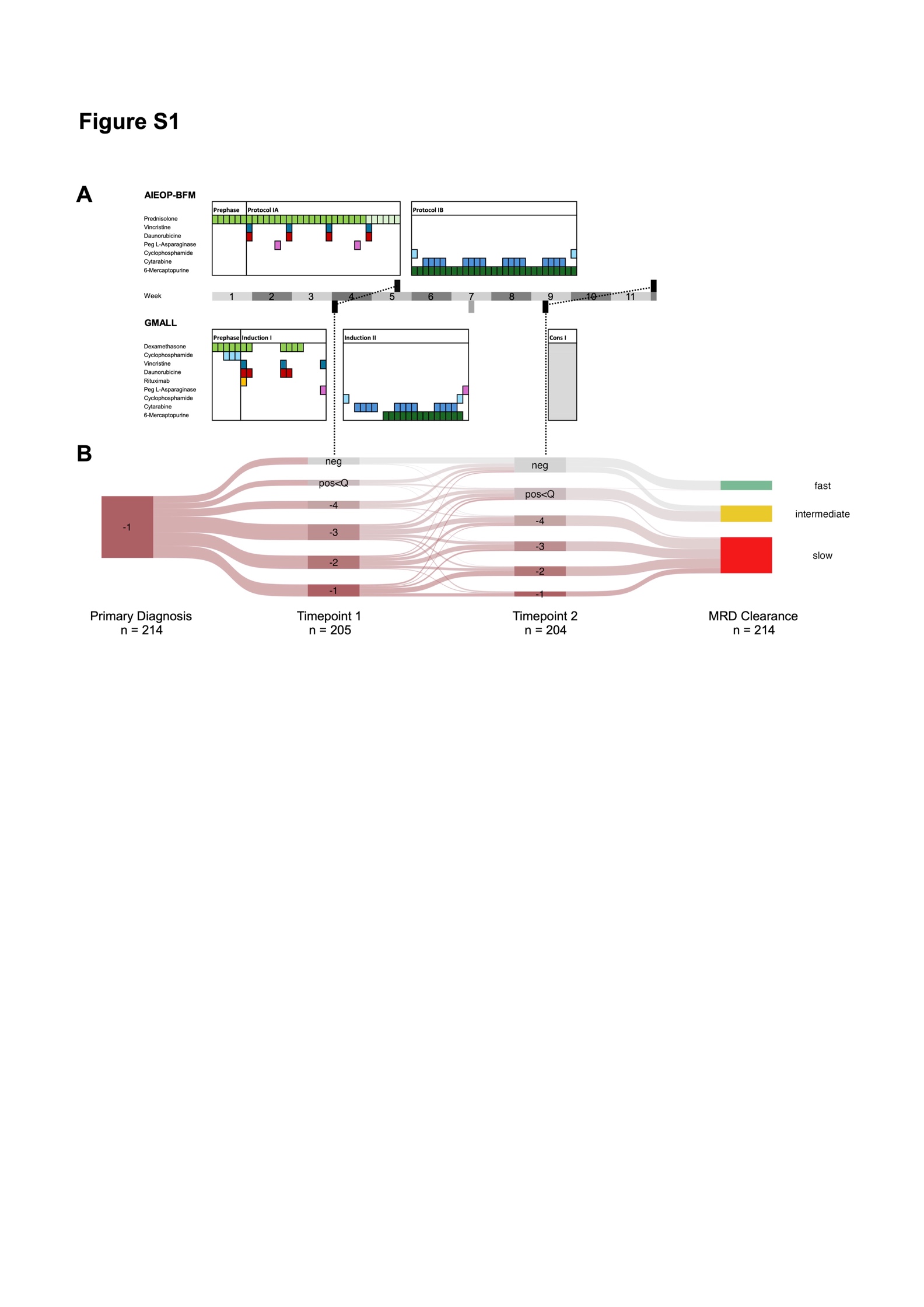


**S2. Maturity score based on ALLCatchR enrichment scores for proximity to normal B-lymphopoiesis.** To calculate maturity scores per patient, enrichment values for each cell type (y-axis) were plotted against relative cell type distances (x-axis), calculated from group distances in a PCA of immuno-genomic defined RNAseq reference samples of human B-lymphopoiesis(1). A linear regression was fitted through the five data points and the slope of each regression curve was defined as the individual maturity score. **A**: PCA of ALLCatchR enrichment scores of seven FACS-sorted healthy B-cell populations from four donors repsectively(1). **B**: Exemplary regression for three samples with low (top panel), intermediate (middle panel) and high (bottom panel) maturity scores. **C**: KMT2A cohort mapped to PCA from (A). each dot represents one patient, colored by maturity score. Patients map along the pseudo-trajectory created in the PCA space according to their maturity score. **D**: KMT2A cohort ALLCatchR enrichment scores for each cell type ordered by the patients maturity score demonstrates the shift of predominant enrichment for early cell stages (Pro-B) in cases with low maturity score towards predominant enrichment in later cell stages (Pre-B II /Immature) in cases with high maturity score.


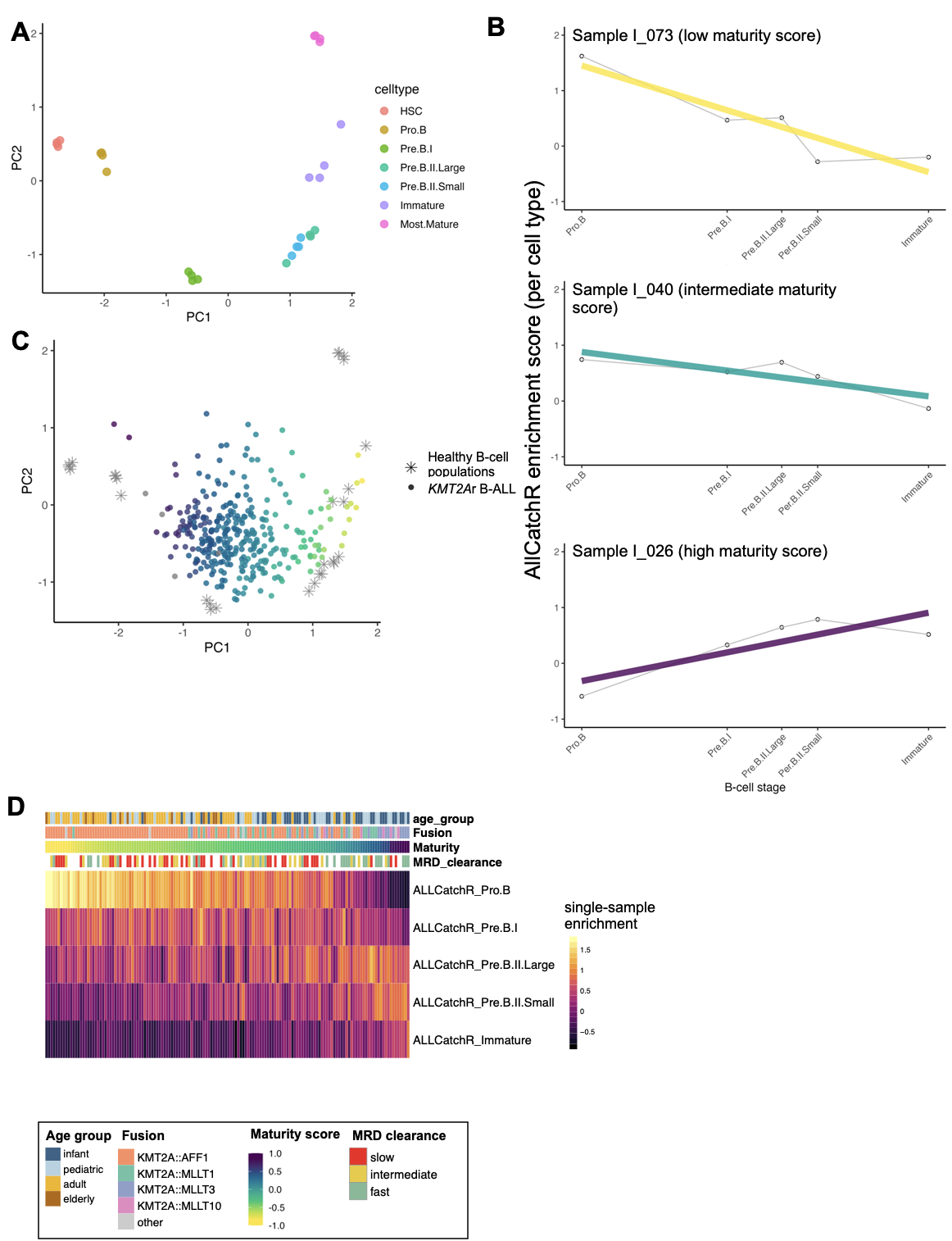


**S3. Validation of maturity score by comparing to multipotency score A:** Per sample maturity score (x-axis) vs. Multipotency Score as postulated by (2) and correlation of scores (Pearson correlation R^2^=0.54, P<0.001). Solid line represents linear regression curve. **B:** Boxplots of per patient maturity scores per predicted B-cell developmental map cell type. **C:** Boxplots of per patient Multipotency scores per predicted B-cell developmental map cell type.


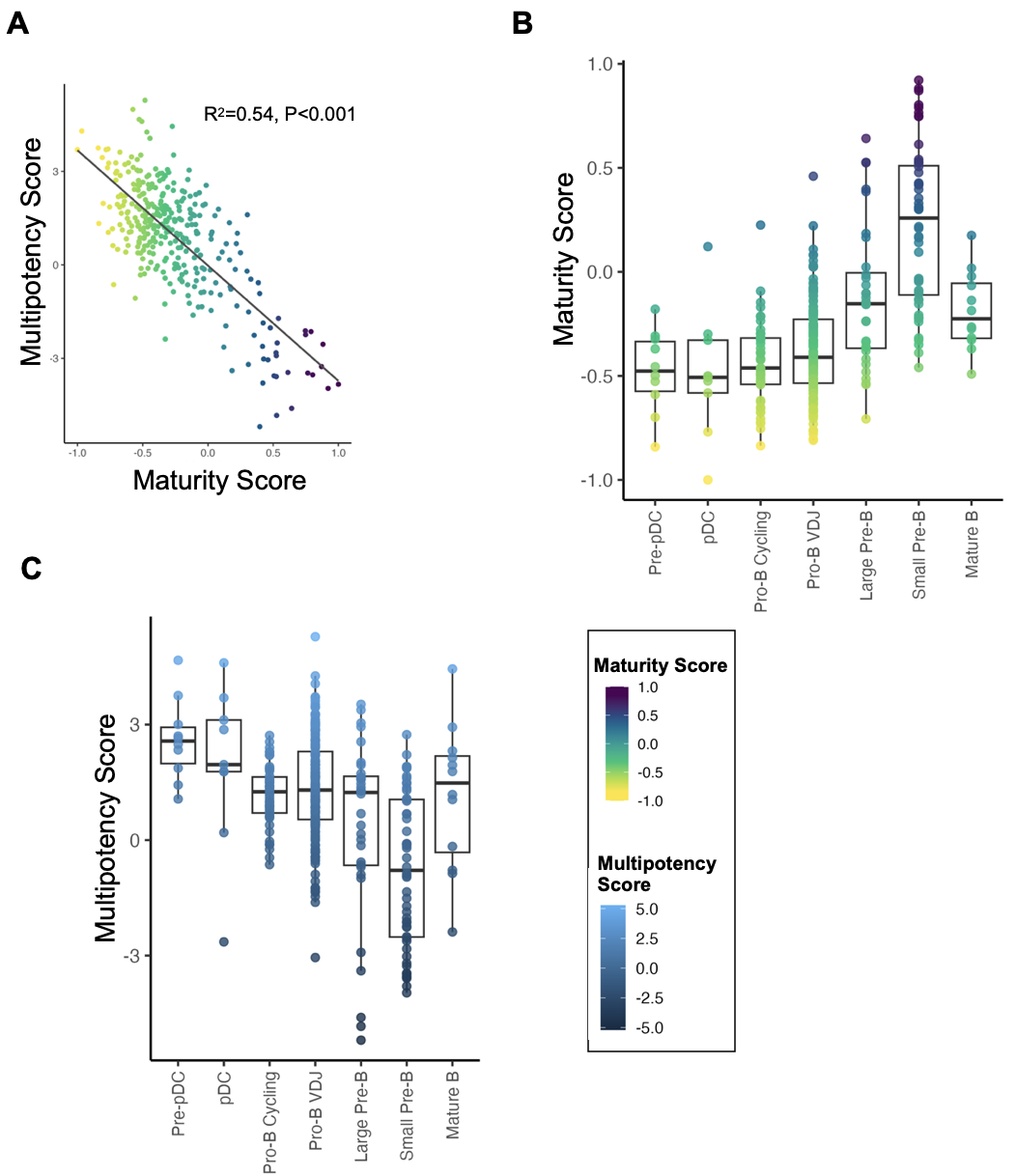


**S4. Validation of maturity score by mapping to human bone marrow map reference atlas. A:** Predicted per sample cell types and projection to umap. Each bulk RNA-sample was projected to the human bone marrow map(3) using the BoneMarrowMap R package(4). Projected samples are colored by maturity score value. **B**: Reference cell types.


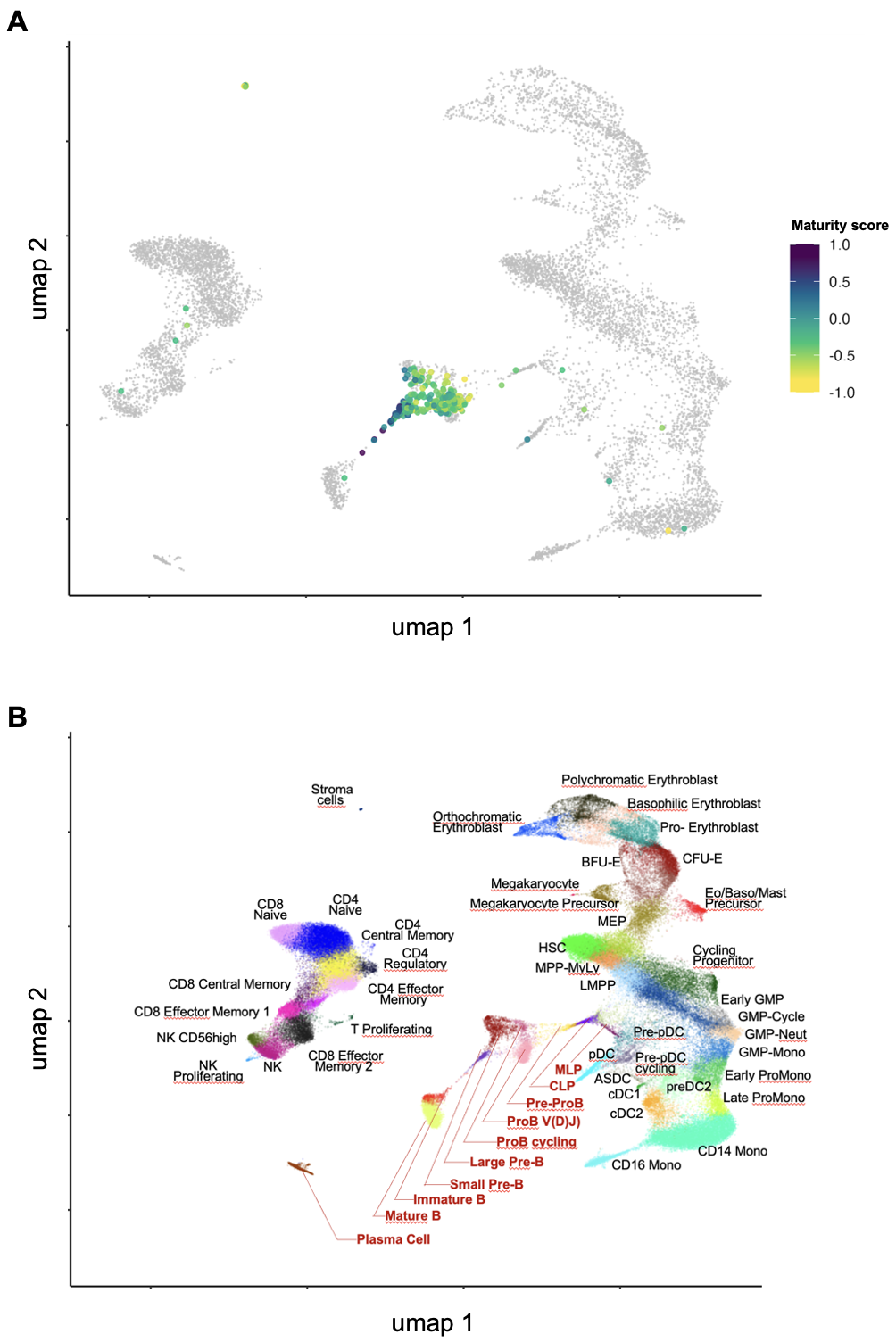


**S5. Clonal evolution from DNA-based IG-R NGS sequencing with respect to maturity score** Maturity scores with respect to presence of clonal evolution from IG-R NGS sequencing (n=47 samples of which n=9 infant, n=9 pediatric, n=16 adult; Wilcox-test p=0.0028).


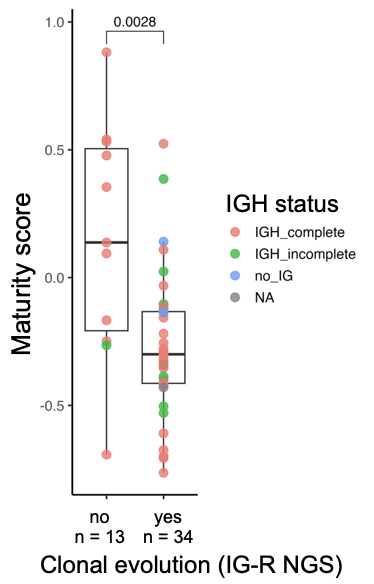


**S6. Unsupervised clustering of top 1000 variably expressed genes in *KMT2A*r B-ALL discovery cohort.** Normalized gene expression was clustered using hclust function implemented in the R package “pheatmap” with “ward.D2” clustering algorithm.

**S7.** **Genomic alterations in *KMT2A*r B-ALL**. **A**: Oncoplot showing mutations detected by the lymphoid DNA capture panel in analyzed samples (n=82 patients: n=14 infant, n=12 pediatric, n=50 adult, n=6 elderly). **B**: Karyotypes from SNParrays (n=104) revealed diploid karyotypes in nearly all cases.


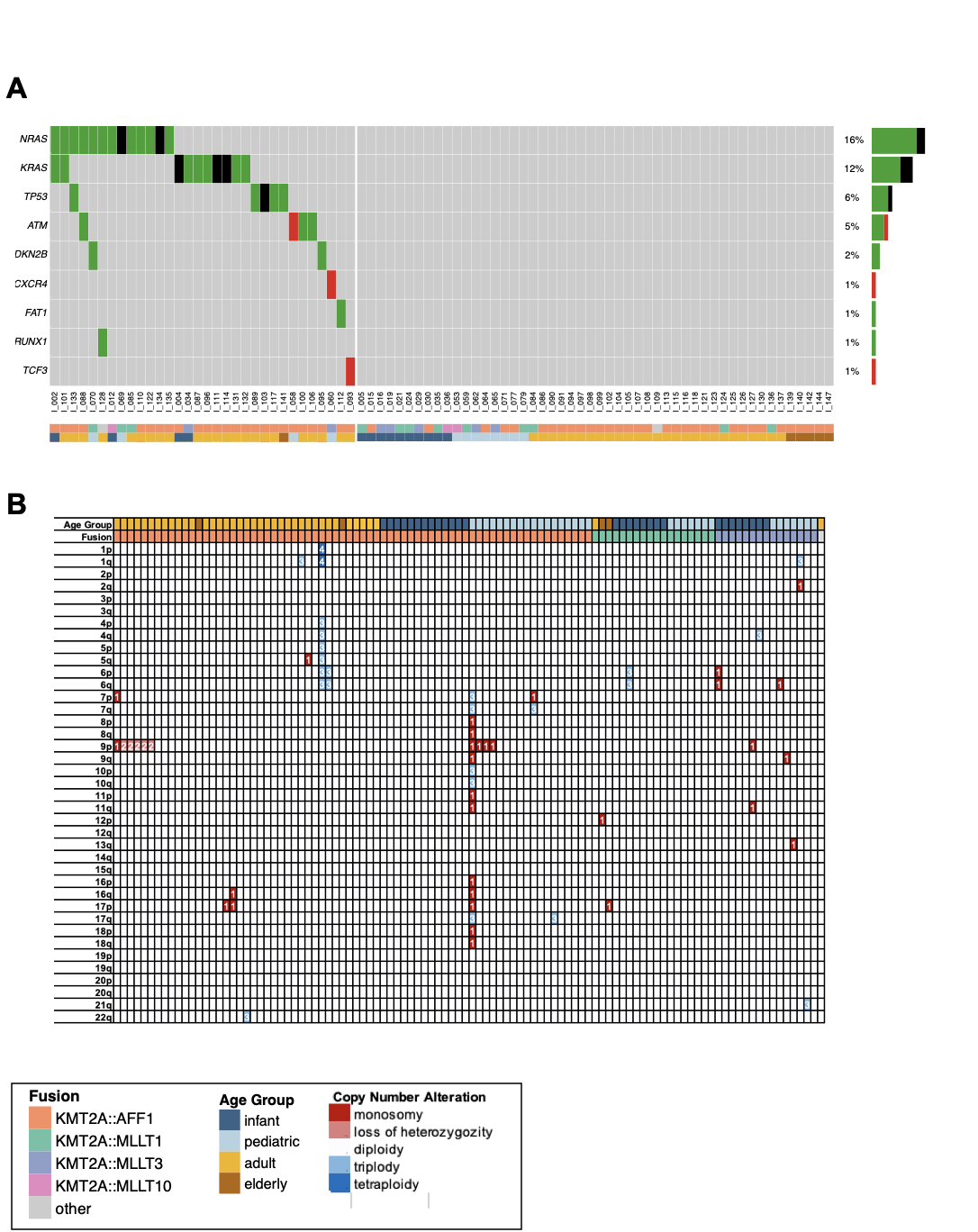


**S8. Maturity score correlates with fusion and age groups and differs between MRD clearance groups.** **A**: Fine-scale pattern of MRD clearance categories per age group and fusion including n=144 *AFF1*r, n=23 *MLLT1*r, n=18 *MLLT3*r and n=28 cases with other or unknown fusion. **B**: Predictor importance and significance in the multivariable logistic regression (Main figure 3C) by drop-1-statistics. For each predictor (WBC, fusion partner, maturity score, age group, sex) a reduced model without that predictor was tested against the full model to assess how much each predictor contributes to the model given the others. Contribution is represented by bar length (delta Deviance) and p-values are annotated for each factor.


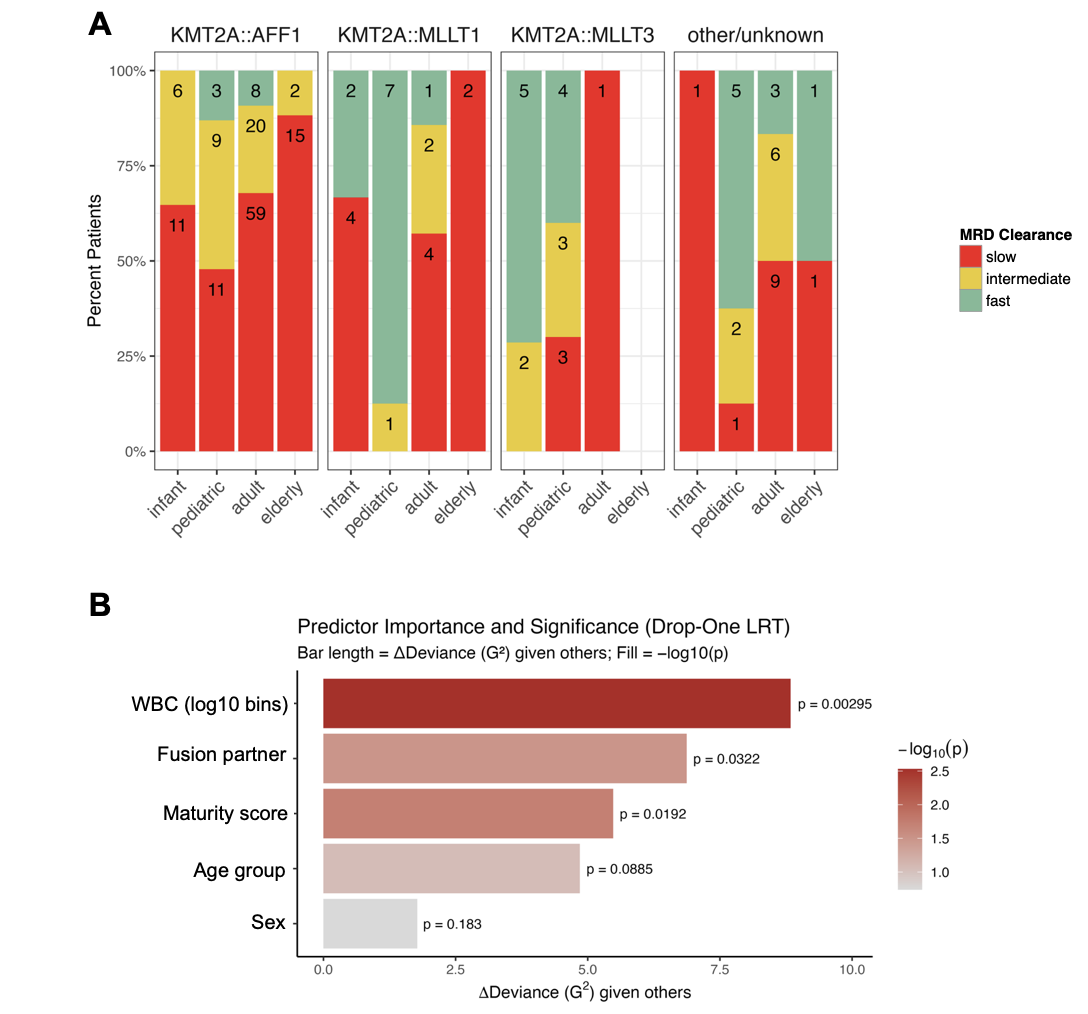


**S9.** **Gene regulatory landscape of *KMT2A*r fusion partners. A:** Heatmap of hierarchical clustering of fusion specific genes detected in supervised analysis using multi-comparison ANOVA and subsequent LASSO feature selection for genes separating *AFF1*r, *MLLT1*r and *MLLT3*r (n=148 samples, n=236 genes). Hierarchical clustering splits *AFF1*r and non-*AFF1*r patients, the second split subdivided non-*AFF1*r cases. **B**: Venn diagram of gene set intersection between MRD score genes, Fusion-specific genes from (A) and genes significantly correlated with age and MRD score genes and genes used in the ALLCatchR tool(1) for proximity scores to normal B-cells. **C**: Gene ontology annotation for genes upregulated in MRD Cluster 3 from main figure 3E. Gene set was annotated with enriched GO terms (GO:Biological Process). Next, enriched GO-terms were filtered for terms size <1000, terms including at least five genes from the gene list and genes were filtered for genes present in at least ten GO-terms. The resulting genes and terms were grouped by functional similarity and five groups were summarized based on included GO-terms.


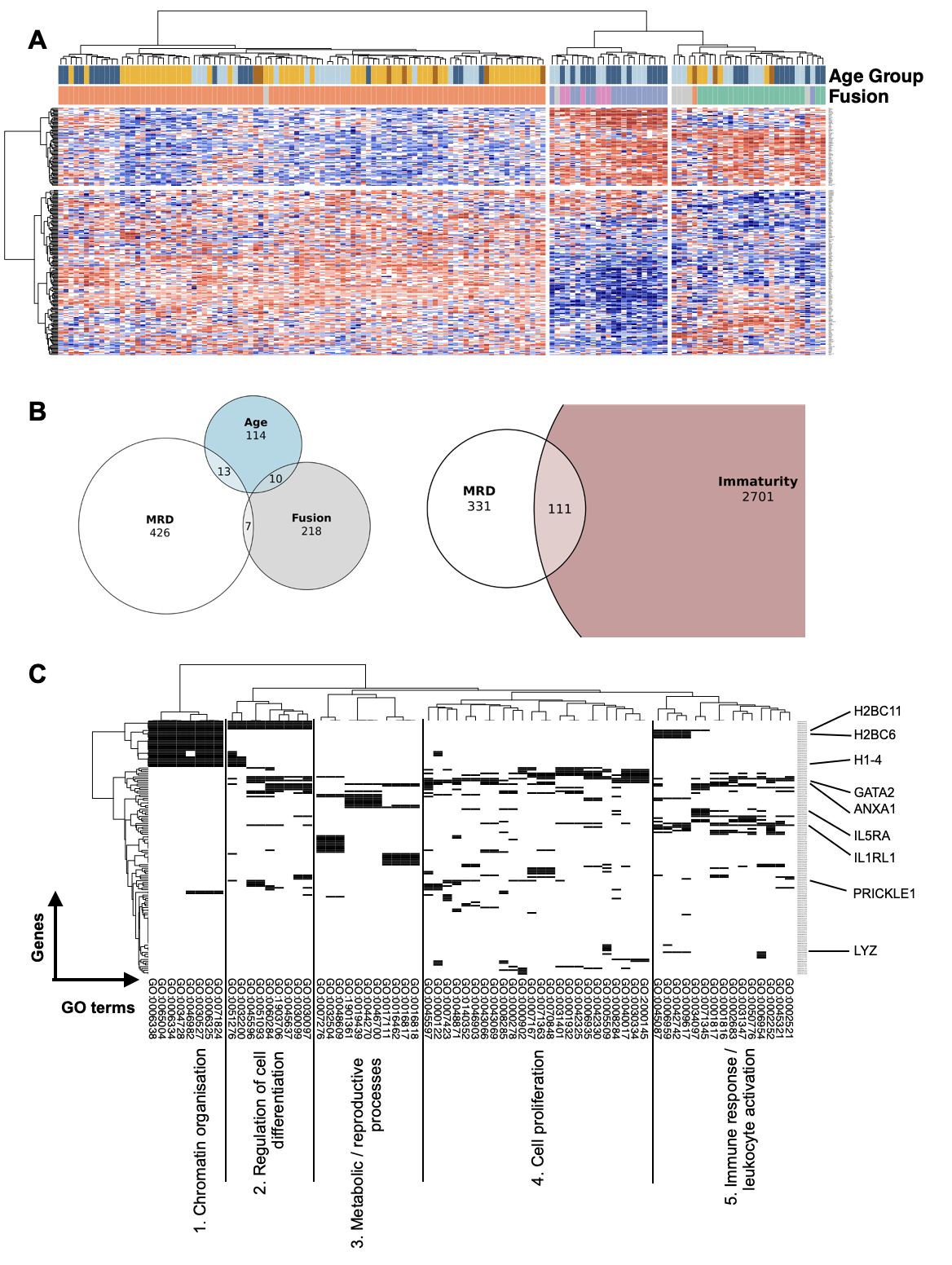


**S10. Enrichment for MRD clearance category, maturity score, fusion and age group per induction phase and Venetoclax response quartile.** Patients were split into quartiles based on mean response to induction phase drugs (Asparaginase, Cytarabine, Daunorubicin, Dexamethasone, Doxorubicin, Vincristine; n=59) or Venetoclax (n=58). Left side: DRP induction phase drugs. MRD clearance (fast vs. intermediate/slow clearance, Q1/2: vs. Q3/4, Fisher’s exact test p=0.004), maturity scores (<=-0.28 (immature); <=-0.04 and >-0.28, >-0.04 (mature), Q1/2 vs. Q3/4 Chi-sq. test p=0.055), fusion partners (*AFF1* vs non-*AFF1* Q1/2: vs. Q3/4, Fisher’s exact test p=0.01), age groups (infant/pediatric vs adult/elderly, Q1/2 vs. Q3/4, Fisher’s exact test p=0.16). Right side: DRP Venetoclax. MRD clearance (fast vs. intermediate/slow, Q1/2 vs. Q3/4, Fisher’s exact test p=0.75), maturity scores (<=-0.28 (immature); <=-0.04 and >-0.28, >-0.04 (mature), Q1/2 vs. Q3/4, Chi-sq. test p=0.41), fusion partners (*AFF1* vs non-*AFF1*, Q1/2 vs. Q3/4, Fisher’s exact test p=0.15) age group (infant/pediatric vs adult/elderly, Q1/2 vs. Q3/4, Fisher’s exact test p=0.25).


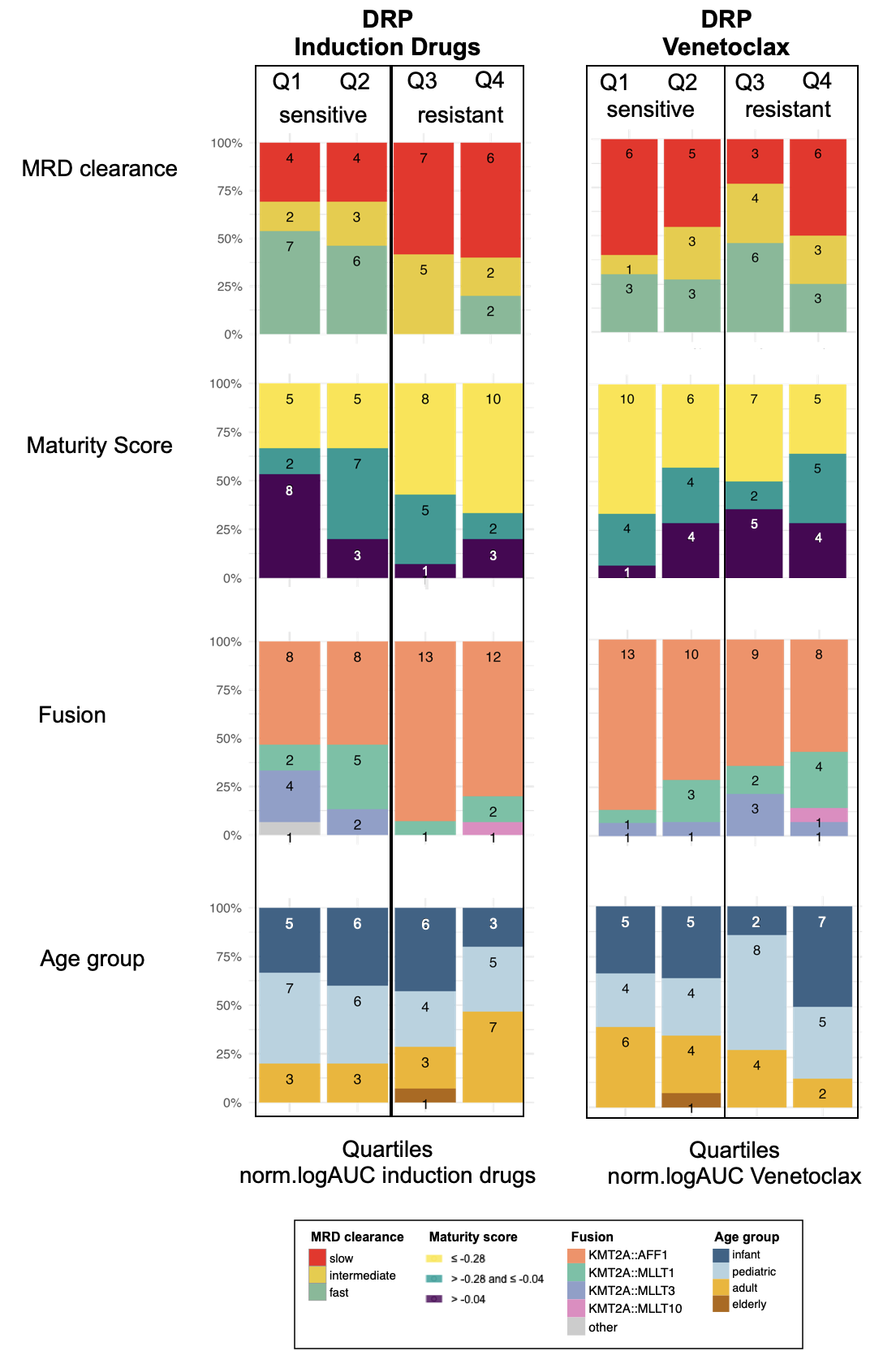


**S11.** **Menin-inhibitors across *KMT2A*r B-ALL subgroups by DRP.** LogAUC values for Menin-Inhibitors MI-503, SNDX-5613 and VTP-50469 with respect to **A:** Fusion (ANOVA p values denoted), **B**: Maturity score (pearson correlation coefficient and p-value noted, solid line represents linear regression), **C:** Age group (ANOVA p-values denoted) and **D:** MRD clearance. Boxes with less than 3 data points were removed.


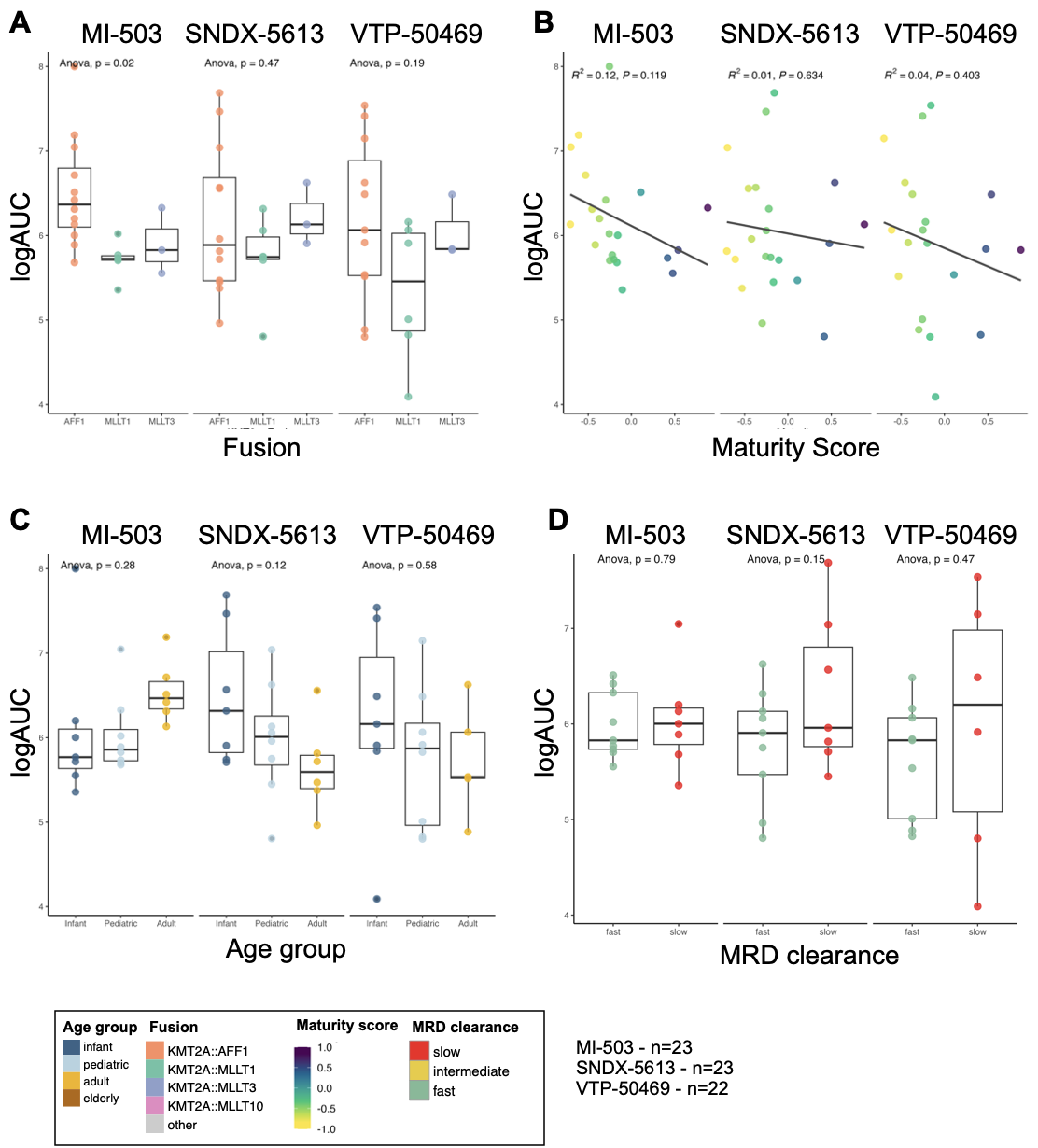


**References Supplementary Figures**

1. Beder T, Hansen BT, Hartmann AM, Zimmermann J, Amelunxen E, Wolgast N, et al. The Gene Expression Classifier ALLCatchR Identifies B-cell Precursor ALL Subtypes and Underlying Developmental Trajectories Across Age. HemaSphere. 2023 Sept;7(9):e939.

2. Iacobucci I, Zeng AGX, Gao Q, Garcia-Prat L, Baviskar P, Shah S, et al. Multipotent lineage potential in B cell acute lymphoblastic leukemia is associated with distinct cellular origins and clinical features. Nat Cancer [Internet]. 2025 June 27 [cited 2025 July 25]; Available from: https://www.nature.com/articles/s43018-025-00987-2

3. Zeng AGX, Iacobucci I, Shah S, Mitchell A, Wong G, Bansal S, et al. Single-cell Transcriptional Atlas of Human Hematopoiesis Reveals Genetic and Hierarchy-Based Determinants of Aberrant AML Differentiation. Blood Cancer Discov. 2025 Apr 28;OF1–18.

4. Zeng AGX. _BoneMarrowMap: Single cell reference mapping onto Bone Marrow Hematopoiesis_. 2024.
